# Metabolic and Cardiovascular Benefits of Apple and Apple-Derived Products: A Systematic Review and Meta-Analysis of Randomized Controlled Trials

**DOI:** 10.3389/fnut.2022.766155

**Published:** 2022-04-05

**Authors:** Sun Jo Kim, Nguyen Hoang Anh, Cheol Woon Jung, Nguyen Phuoc Long, Seongoh Park, Young Hyun Cho, Young Cheol Yoon, Eun Goo Lee, Mina Kim, Eui Young Son, Tae Ha Kim, Yingqian Deng, Johan Lim, Sung Won Kwon

**Affiliations:** ^1^College of Pharmacy, Seoul National University, Seoul, South Korea; ^2^Research Institute of Pharmaceutical Sciences, Seoul National University, Seoul, South Korea; ^3^Department of Statistics, Sungshin Women’s University, Seoul, South Korea; ^4^Department of Statistics, Seoul National University, Seoul, South Korea; ^5^Plant Genomics and Breeding Institute, Seoul National University, Seoul, South Korea

**Keywords:** placebo-controlled study, metabolic syndrome, cardiovascular disease, apple polyphenol, apple pectin, blood marker, lipid

## Abstract

**Background:**

Quantitative evidence of the metabolic and cardiovascular effects of apples (*Malus domestica*) is lacking in interventional studies. This study aimed to summarize the available evidence of the beneficial effects of apples and apple-derived products (ADPs) on metabolic and cardiovascular markers.

**Methods:**

Peer-reviewed randomized controlled trials (RCTs) were identified from four databases on May 3, 2021 and regularly updated until the end of May 2021. Demographic characteristics, intervention types, and evaluation parameters were extracted. A meta-analysis on the mean difference of change scores was conducted on commonly presented outcomes in the RCTs.

**Results:**

The metabolic and cardiovascular effects of diverse regimens, including whole apple, apple extract, and apple juice, were examined in 18 eligible RCTs. Nine common evaluation outcomes were eventually introduced to the meta-analysis, including total cholesterol (TC), low-density lipoprotein (LDL), high-density lipoprotein (HDL), triglyceride, glucose, insulin, C-reactive protein, and systolic/diastolic blood pressures. The levels of TC (−2.69 mg/dL; 95% CI: −5.43, 0.04 mg/dL) and LDL (−2.80 mg/dL; 95% CI: −5.78, 0.17 mg/dL) showed a non-significant decreasing tendency after at least a week of apple consumption. Further subgroup analysis, particularly, a comparison with placebo as a control, showed a significant reduction in TC and LDL levels. When stratified by the baseline level, subjects with high TC and LDL level were shown to have more benefits from the apple intake. Intriguingly, apple and ADPs significantly reduced HDL levels to a small extent (−1.04 mg/dL; 95% CI: −1.79, −0.29 mg/dL). The other markers were mostly unaffected by the intervention.

**Conclusion:**

Our investigation revealed that apples could improve blood cholesterol levels.

**Systematic Review Registration:**

[https://www.crd.york.ac.uk/prospero/], identifier [CRD42020215977].

## Introduction

The apple (*Malus domestica*) is one of the most widely consumed fruits and accounts for 12.5% of all fruits worldwide (1). Approximately 26.9 million small fresh apples are consumed daily in the United States (2) and approximately 22 kg of apples is consumed per capita annually in the total European diet (3). The functional properties of apples on metabolic syndromes have steadily gained attention, particularly because they are a rich source of functional phytochemicals such as flavonols, phloretin, and procyanidin oligomers (4–7). More specifically, apples and apple-derived products (ADPs) exert anti-obesity activities and reduce the risk of cardiovascular diseases (6–8). As a result, several systematic reviews have been conducted on the evidence-based outcomes of the biological effects of apple intake on metabolic and cardiovascular markers and complications. For example, a meta-analysis of prospective cohort studies on type 2 diabetes reported on the beneficial effect of apples on reducing the risk of disease (9). Notably, two systematic reviews evaluated the alteration of metabolic parameters following apple (10) or apple vinegar intake (11). Nevertheless, these studies were either limited to observational studies or lacked statistical power due to the small sample size.

This study aimed to systematically analyze randomized controlled trials (RCTs) that investigated the clinical functions of apples and ADPs through oral intake and evaluate their effects on the improvement of metabolic and cardiovascular markers compared to placebo or any alternative diet. There was no limitation regarding the processing method of ADPs, and all human subjects were considered eligible regardless of age and basal condition. Finally, we deduced the effect of apple and ADP intake on the nine most commonly reported markers by conducting a meta-analysis with exhaustive subgroup analyses.

## Methods

### Search Strategy

The study was registered at the PROSPERO International Prospective Register of Systematic Reviews (CRD42020215977) and followed the Preferred Reporting Items for Systematic Reviews and Meta-Analyses guidelines (Supplementary Table 1) (12). The databases PubMed, Embase, CENTRAL, and ClinicalTrials.gov were systematically searched with terms related to “apple” and metabolic and cardiovascular biomarkers such as “lipid profile,” “glucose,” “insulin,” and “C-reactive protein.” The full search terms are listed in Supplementary Table 2. There were no limitations in terms of the search period and language of the publications. Articles were preliminarily searched on February 13, 2020 and were first collected on May 3, 2021. The list was regularly updated by searching for RCTs published between the initial search date and the end of May 2021. Duplicates were automatically removed in Endnote X9, followed by manual inspection by two reviewers (SJK and NA) in a blinded manner.

### Study Selection

We collected peer-reviewed RCTs that investigated the effects of oral intake of apple or ADPs on metabolic and cardiovascular markers. The markers included circulating lipids and lipoproteins, C-reactive protein (CRP), diabetic indicators, and blood pressure. We considered an RCT to be eligible for meta-analysis only if it provided quantitatively synthesizable data for calculating the mean difference of change score between the apple and control groups.

A study was deemed ineligible for inclusion if it was (did) (i) not designed as an RCT; (ii) unrelated to apple intake; (iii) not able to provide relevant data, such as the study employed significant apple intake as a control treatment or a comparison among different types of apple species; (iv) investigate the acute effect of apple intake (less than a week of treatment); or (v) review, conference, abstract, or any other secondary scientific reports. At least three reviewers (SJK, NA, and CWJ) independently evaluated the eligibility of each study in a blinded manner by screening the titles and abstracts. Disagreements were resolved by a third party through a careful discussion. Additional screening of reference reviews was performed to improve the coverage. We employed Rayyan QCRI, an open-source repository, to facilitate the screening of abstracts and titles (13).

### Data Extraction

All data extraction procedures were performed by at least two reviewers for each RCT, and consensus was reached via discussion when conflicts arose. We first collected demographic information, including cohort allocation (country), basal disease condition, sex, and sample size. In terms of experimental details, study design (crossover vs. parallel), dosage, type of product, evaluation system, and adverse effects were recorded if applicable. Additionally, we conducted an in-house quality control, where another reviewer randomly checked the extracted information without any prior discussion.

### Meta-Analysis

A meta-analysis was conducted using Review Manager 5.4 (Nordic Cochrane Center, The Cochrane Collaboration version 5.4). Common outcome parameters across the included studies were screened. An outcome was considered suitable for meta-analysis when more than five studies reported the outcome by either a change score or both baseline and post-intervention values with a treatment duration of at least 1 week. Therefore, we focused on the changes in total cholesterol (TC), high-density lipoprotein (HDL), low-density lipoprotein (LDL), triglyceride (TG), glucose, insulin, CRP, systolic blood pressure (SBP), and diastolic blood pressure (DBP). Before data integration, the units of TC, HDL, LDL, and TG data were converted to milligram per deciliter (mg/dL) by multiplying mmol/L by 38.67 for TC, HDL, and LDL, and by multiplying mmol/L by 88.57 for TG. In addition, the unit for glucose was converted from mmol/L to mg/dL by multiplying by 18.02, while the unit for insulin was converted to μU/mL. From individual studies, we extracted information on the change scores from baseline to post-intervention and considered their differences from the treatment and control groups. The standard error (*SE*) values of the differences were also determined. If a study did not provide the necessary statistics, we manually calculated them, as explained. The difference in the change scores was easily determined by subtracting the mean values of the treatment and control groups. However, the calculation of *SE* differed depending on the type of information (change in score vs. mean/standard deviation) and the type of experiment (parallel vs. crossover). To provide a more explicit explanation, we introduce the notations in Table 1. The calculation formulas used to derive the *SE* for four different cases are summarized in Table 2. For example, ρ_*X*_1_,*X*_2__ is the correlation coefficient between the two states *X*_*1*_ and *X*_*2*_. Notably, the *SE* of a crossover study was derived with an additional assumption of *X*_2_ = *Y*_2_, which stems from the assumption that a washout period eliminates the effect of intake in an individual. All statistics, except for the correlation coefficient(s), were obtained from a study depending on the type of given information and experiment. We set unknown correlation coefficients as 0.5, which is in line with previous literature (14). To check for potential bias from the imputed correlation, sensitivity analysis was conducted by plugging in 0.2 and 0.8 as the correlation coefficients. For a study with multiple comparison groups, the most relevant intervention and control groups were chosen (e.g., whole apple vs. placebo, instead of whole apple vs. vitamin juice). In addition, if a study reported measurements at multiple time points, then the time point most approximate to other studies was used (e.g., 4 weeks of intervention). Studies that reported only percentage changes as results and studies with results that could not be used due to missing data were excluded from the meta-analysis. Once all information was collected from the studies, we analyzed the input values using a generic inverse variance outcome and displayed the data as mean difference (MD) with a 95% confidence interval (CI) for measuring the effect for all the outcomes, except CRP, for which the standardized mean difference (SMD) of change scores was applied because there was high heterogeneity regarding the magnitude of blood levels. Data transformation from MD and *SE* of MD to SMD and *SE* of SMD was performed according to the type of experiment (parallel vs. crossover) for each study, following Cochrane guidelines (15, 16). We used a random-effects model because it incorporates both within-study and between-study heterogeneity. The effects of apples and ADPs were visualized using forest plots. Cochran’s *Q* test and *I*^2^ statistics were used to inspect heterogeneity across the studies. Multiple subgroup analyses were performed based on the participants’ basal condition (healthy subjects and subjects with metabolic and cardiovascular risk), treatment intervention type (whole apple, apple polyphenol, apple pectin, and apple vinegar), control intervention type (placebo and other materials), baseline level of the marker (low and high), sample type (serum and plasma), and design of the study (crossover and parallel). Placebo here indicates control diet treated with either true placebo that is undistinguishable to apple intervention or no intervention, without any specific functional compounds. Furthermore, a leave-one-out sensitivity analysis was performed to examine whether the overall effect depended heavily on a specific study. Statistical significance was set at *p* < 0.05.

**TABLE 1:** Notations for characteristic value.

	Control		Apple	
	Before intake	After intake	Before intake	After intake
Observation	*X*_2_	*X*_1_	*Y*_2_	*Y*_1_

Sample size	*N*_*X*_2__	*N*_*X*_1__	*N*_*Y*_2__	*N*_*Y*_1__
	
	*N*_*X*_, if *N*_*X*_1__ = *N*_*X*_2__		*N*_*Y*_, if *N*_*Y*_1__ = *N*_*Y*_2__	

Standard deviation	σ_*X*_2__	σ_*X*_1__	σ_*Y*_2__	σ_*Y*_1__

Difference	*d*_*X*_ = (*X*_1_−*X*_2_)		*d*_*Y*_ = (*Y*_1_−*Y*_2_)	

Standard deviation of difference	σ_*d*_*X*__		σ_*d*_*Y*__	

**TABLE 2:** Calculation of standard error based on the type of information (change score vs. mean/standard deviation) and the type of experiments (parallel vs. crossover).

*SE*	Change score	Mean/standard deviation
Parallel	$\sqrt{\frac{\sigma_{d_{X}}^{2}}{N_{X}}+\frac{\sigma_{d_{Y}}^{2}}{N_{Y}}}$	$\sqrt{\frac{\sigma_{X_{1}}^{2}}{N_{X_{1}}}+\frac{\sigma_{X_{2}}^{2}}{N_{X_{2}}}-2\,\frac{\rho_{X_{1},X_{2}}\,\sigma_{X_{1}}\,\sigma_{X_{2}}}{\max\left(N_{X_{1}},N_{X_{2}}\right)}+\frac{\sigma_{Y_{1}}^{2}}{N_{Y_{1}}}\,\frac{\sigma_{Y_{2}}^{2}}{N_{Y_{2}}}-2\,\frac{\rho_{Y_{1},Y_{2}}\,\sigma_{Y_{1}}\,\sigma_{Y_{2}}}{\max\left(N_{Y_{1}},N_{Y_{2}}\right)}}$
Crossover	$\sqrt{\frac{\sigma_{d_{X}}^{2}}{N_{X}}+\frac{\sigma_{d_{Y}}^{2}}{N_{Y}}-2\,\frac{\rho_{d_{X},d_{Y}}\,\sigma_{d_{X}}\,\sigma_{d_{Y}}}{\max\left(N_{X},N_{Y}\right)}}$	$\sqrt{\frac{\sigma_{X_{1}}^{2}}{N_{X_{1}}}+\frac{\sigma_{Y_{1}}^{2}}{N_{Y_{1}}}-2\,\frac{\rho_{X_{1},Y_{1}}\,\sigma_{X_{1}}\,\sigma_{Y_{1}}}{\max\left(N_{X_{1}},N_{Y_{1}}\right)}}$

*ρ_A,  B_, correlation coefficient between A and B states.*

### Publication Bias

Publication bias was evaluated for every marker using funnel plot visualization. Egger’s regression test and Begg’s rank test (not-continuity corrected) were conducted to determine the statistical values. A *p*-value of less than 0.1 indicated the existence of publication bias. Publication bias assessment was not performed for insulin and CRP because of the small number of studies (less than 10).

### Quality Assessment

The risk of bias in individual studies was evaluated using the latest Cochrane Collaboration tool (RoB 2) for randomized trials (17). The tool introduces five distinct domains to assess different sources of bias, with each domain having multiple signaling questions. Individual domains were independently evaluated based on the answers to their signaling questions and categorized as either “low risk of bias,” “some concerns,” or “high risk of bias.” Multiple reviewers assessed the methodological qualities of all the included studies, and a consensus was reached via discussion of the conflicting judgments.

### Quality of Evidence

The quality of evidence of meta-analysis outcomes was rated using the Grading of Recommendations Assessment, Development, and Evaluation (GRADE) (18). Since the current meta-analysis was conducted on RCTs, all evidences began as high-quality and gradually downgraded following the number of drawbacks, including risk of bias, inconsistency, indirectness, imprecision, and publication bias.

## Results

### Search Results

**FIGURE 1:**
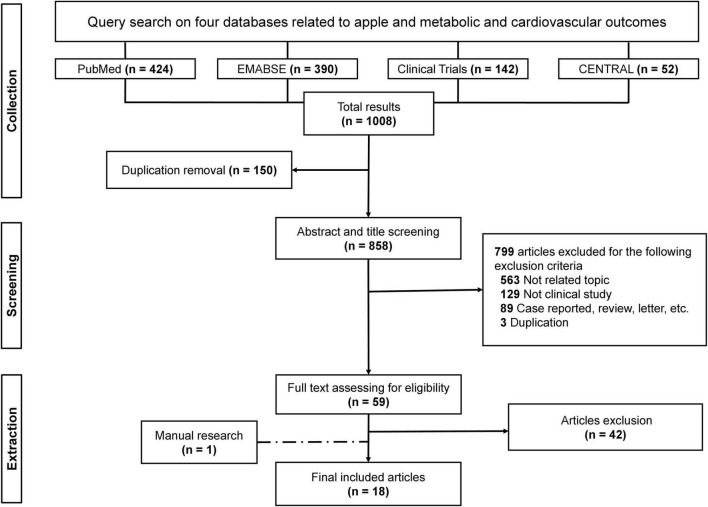
Flowchart of the study selection process.

After performing systematic searches on the four databases, we retrieved 1,008 studies in total. Duplication was checked after all studies were imported into Endnote X9, and 150 duplicates were removed. Thereafter, 858 studies were screened for titles and abstracts. After careful evaluation, 799 studies were excluded owing to ineligibility. Finally, 59 studies were gathered for full-text screening. Eighteen studies, including a paper published after the first literature searched (19), were deemed eligible for data extraction. Figure 1 shows the flowchart of the study selection process.

### Study Design and Participants

Among the included RCTs, 11 employed a parallel design (19–29), whereas the others employed a crossover study design (1, 30–35). The overall intervention period ranged from 1 to 12 weeks. Apples were administered in various forms such as in a drink (liquid supplement, clear juice, or puree), whole (fresh or dried), powder, extract, vinegar, and pomace. The apple extract was the most frequently tested ADP, particularly reinforced in polyphenolic compounds and apple pectin. Two other common forms of administration were unprocessed whole apple and apple juice. Whole-apple intake ranged from one to three apples per day. Cohort allocation was mainly located in Europe and North America.

### Total Cholesterol, High-Density Lipoprotein, Low-Density Lipoprotein, and Triglyceride

Blood lipid profiles, including TC, HDL, LDL, and TG, were the most frequently reported metabolic markers following apple or ADP intake. Among the included RCTs, 17 (*n* = 793) provided applicable data for meta-analysis (1, 19–27, 29–35). Among them, 15 (*n* = 670) reported four lipid profile parameters; one study reported HDL, LDL, and TG (32); and the remaining one reported HDL and TG as the change scores (20). Regarding study design, 10 studies used a parallel design (19–27, 29), while the other seven employed a crossover design (1, 30–35). Regarding the type of intervention, apple polyphenols (*n* = 9) (21, 23, 25, 29–31, 33–35) were the most frequently used, followed by whole apple (*n* = 6) (1, 19, 20, 22, 27, 32), and apple pectin (*n* = 2) (24, 26). The treatment dosage varied depending on the type of formulation used. Serum and plasma were the two types of samples collected for measuring lipid levels in all trials. Eleven studies used placebo diets for control intervention (1, 19, 21, 23–27, 29, 33, 35), while the other studies used another type of intervention or a habitual diet, which might possess a different extension of the effect. Most of the included trials were conducted on a population with cardiovascular disease (CVD) risk factors (*n* = 13) such as dyslipidemia, high blood pressure, and obesity (1, 19, 21–25, 27, 29, 31, 33–35). The intervention period ranged from 1 to 12 weeks, with 4 weeks being the most common. The data on the study characteristics and evaluation parameters are presented in Table 3.

**TABLE 3:** Summarized information of the studies which were incorporated into the meta-analysis.

Author, allocation, year	Type of randomized controlled trial	Basal condition	Intervention			Control		Intervention period	Evaluation system	Adverse effect
			M/F	Administration	Formula of apple	M/F	Administration			
Gasper et al. (30) United Kingdom	Crossover	Healthy	13/12	230 g of high flavanol-containing apple puree intervention (about 100 mg epicatechin)/day	Puree	13/12	Aspirin intervention (75 mg)/day	4 weeks	Serum CRP, serum TC, serum HDL, serum LDL, serum TG, serum endothelin-1, plasma nitric oxide metabolites	N/A
			13/12	230 g of low flavanol-containing apple puree intervention (about 25 mg epicatechin)/day	Puree					
Chai et al. (20) United States	Parallel	Healthy postmenopausal female	0/45	75 g of dried apple/day	Dried apple	0/55	Dried plum	1 year	Serum TC, serum LDL, serum HDL, serum TG, serum CRP, serum lipid hydroperoxide, BMI	N/A
Cicero et al. (21) Italy	Parallel	Overweight	15/16	300 mg of apple polyphenols/day	Powder	16/15	300 mg of placebo	8 weeks	Serum TC, serum HDL, serum LDL, serum TG, serum VLDL, fasting glucose, apolipoprotein A1, apolipoprotein B, creatine phosphokinase, SBP, DBP, PP, endothelial reactivity	No
Vafa et al. (22) Iran	Parallel	Hyperlipidemia and overweight male	23/0	300 g of golden delicious apple	Whole	23/0	Regular dietary	8 weeks	Serum TC, serum HDL, serum LDL, serum TG, serum VLDL, apolipoprotein B, lipoprotein a	N/A
Akazome et al. (23) Japan	Parallel	Healthy with 25 ≤ BMI < 30	28/19	340 g beverage containing 600 mg of apple polyphenols/day	Drink	28/19	340 g beverage of placebo	12 weeks	Abdominal fat area, body weight, BMI, serum TC, serum HDL, serum LDL, serum TG, non-esterified fatty acids, Remnant-like particle-cholesterol, SBP, DBP, PP	No
		Healthy with 18 ≤ BMI < 30	10/5	1,020 g beverage containing 1,800 mg of apple polyphenols/day	Drink	10/5	1,020 g beverage of placebo	4 weeks		
Bondonno et al. (31) Australia	Crossover	Healthy with cardiovascular risk	10/20	High-flavonoid apple containing total phenolic compounds about 306 mg/day	Whole	10/20	Low-flavonoid apple containing total phenolic compounds about 92 mg/day	4 weeks	SBP, DBP, heart rate, pulse wave velocity, central diastolic pressure, nitrite, nitrate, plasma TC, plasma HDL, plasma LDL, plasma glucose	No
Sirtori et al. (24) Italy	Parallel (2 × 2)	Moderate hypercholesterolemia	11/14	Casein/apple pectin with total mass 152 g/day	Bar	10/15	Casein/cellulose with total mass 152 g/day	4 weeks	Plasma TC, plasma HDL, plasma LDL, plasma TG, glucose, insulin, HOMA-IR, adiponectin, sICAM-1, hs-CRP	Yes^1^
			11/14	Pea protein/apple pectin with total mass 121 g/day	Bar	14/11	Pea protein/cellulose with total mass 121 g/day			
Ravn-Haren et al. (32) Denmark	Crossover	Healthy	9/14	Whole apple 550 g/day	Whole	9/14	No supplement	4 weeks	SBP, DBP, heart rate, weight, plasma TC, plasma HDL, plasma LDL, plasma TG, bile acid concentration, hs-CRP, insulin, insulin-like growth factor 1, IGF binding protein 3	N/A
			9/14	Apple pomace 22 g/day	Pomace					
			9/14	Clear apple juice 500 mL/day	Juice					
			9/14	Cloudy apple juice 500 mL/day	Juice					
Hollands et al. (33) United Kingdom	Crossover	Healthy with moderately elevated blood pressure	15/27	Apple extract containing 70 mg of monomeric flavanols and 65 mg of procyanidins	Powder	15/27	Microcrystalline cellulose	4 weeks	SBP, DBP, serum TC, serum HDL, serum LDL, serum TG, glucose, fructosamine, insulin, endothelin, NO metabolite, augmentation index, aortic SBP, brachial-ankle pulse-wave, carotid femoral pulse-wave velocity, peripheral DBP, peripheral SBP	No
			15/27	Apple extract containing 140 mg of monomeric flavanols and 130 mg of procyanidins	Powder					
			15/27	Apple extract with 130 mg of procyanidins	Powder					
Barth et al. (25) Germany	Parallel	Obesity, non-diabetic male	38/0	750 mL of polyphenol-rich cloudy apple juice (802.5 mg polyphenols)/day	Juice	38/0	750 mL of control beverage (isocaloric)/day	4 weeks	Body weight, BMI, body fat, lean body mass, waist circumference, plasma TC, plasma HDL, plasma LDL, plasma TG, non-esterified fatty acids, adipokines, CRP, sICAM-1, sVCAM-1	N/A
Auclair et al. (34) France	Crossover	Hypercholesterolemia, male	30/0	Polyphenol-rich apple (1.43 g of polyphenol)/day	Powder	30/0	Low polyphenol apple (214 mg of polyphenol)/day	4 weeks	NO metabolite, SBP, DBP, PP, glucose, serum CRP, serum TC, serum HDL, serum LDL, serum TG, apolipoprotein A1, apolipoprotein B	N/A
Koutsos et al. (1) Italy	Crossover	Healthy	18/25	340 g of apple/day	Whole	18/25	500 mL of sugar-matched apple control beverage	8 weeks	Serum TC, serum HDL, serum LDL, serum TG, serum glucose, serum insulin, non-esterified fatty acid, bile acid, anthropometrics (BMI, percent of body fat), VCAM-1, ICAM-1, SBP, DBP, PP	No
Shoji et al. (26) Japan	Parallel	Borderline hyperglycemia	25/7	600 mg of apple polyphenols/day	Powder	22/11	600 mg of starch decomposition product/day	12 weeks	Fasting plasma glucose, fasting plasma insulin, plasma TC, plasma HDL, plasma LDL, plasma TG, cytokine, HbA1c, OGTT, HOMA-IR	N/A
Eisner et al. (27) United States	Parallel	Overweight and obese children	19	Dried apple (240 kcal)/day	Whole	19	Muffins (240 kcal)/day	8 weeks	Fat mass, fat-free mass, body fat composition, serum TC, serum HDL, serum LDL, serum TG, serum glucose, serum insulin, proinsulin, HOMA-IR, serum CRP, adiponectin	N/A
Saarenhovi et al. (35) Finland	Crossover	Borderline hypertension or mild hypertension	26/31	330 mg of apple polyphenol extract/day (containing 100 mg of epicatechin) after 10 h of fasting	Capsulated extract	26/31	335 mg of placebo (microcrystalline cellulose) /day after 10 h of fasting	4 weeks	SBP, DBP, circulating biomarkers (sE-selectin, sVCAM-1, sICAM-1, vWF, ADMA, PAI-1, CRP), fasting plasma TC, fasting plasma HDL, fasting plasma LDL, fasting plasma TG	Yes^2^
Gheflati et al. (28) Iran	Parallel	Type 2 diabetes mellitus and dyslipidemia	10/22	20 mL of apple vinegar/day	Vinegar	10/20	N/A	4 weeks	Fasting blood glucose, HOMA-IR, HOMA-B, QUICKI, serum insulin, SBP, DBP	N/A
Velliquette et al. (29) United States	Parallel	Obese and overweight	7/8	6 g of apple skin extract (80% polyphenol, 5% phlorizin)/day	Capsulated extract	9/8	6 g of placebo	1 week	Serum TC, serum HDL, serum TG, serum LDL	Yes^3^
Liddle et al. (19) Canada	Parallel	Obese and overweight	7/15	Three whole apples (600 g)/day	Whole	7/15	No supplement	6 weeks	Plasma CRP, plasma IFN-γ, plasma glucose, plasma insulin, plasma non-esterified fatty acids, plasma TC, plasma LDL, plasma HDL, HOMA-IR	No

*N/A, not available; VLDL, very low density lipoproteins; BMI, body mass index (kg/m^2^); HOMA-IR, homeostasis model assessment for insulin resistance; sICAM-1, soluble intercellular adhesion molecule-1; hs-CRP, high-sensitivity C-reactive protein; OGTT, oral glucose tolerance test; sVCAM-1, soluble vascular cellular adhesion molecule-1; vWF, von Willebrand factor; ADMA, asymmetric dimethylarginine; PAI-1, plasminogen activator inhibitor-1; HOMA-B, homeostasis model assessment for b-cell function; QUICKI, quantitative insulin sensitivity check index; TC, total cholesterol; LDL, low-density lipoprotein; HDL, high-density lipoprotein; TG, triglycerides; SBP, systolic blood pressure; DBP, diastolic blood pressure; PP, pulse pressure.*

*^1^Nausea, vomiting and minor gastrointestinal side effects.*

*^2^Nasopharyngitis, dyspepsia, headache, migraine, dizziness, ligament sprain, and gamma-glutamyltransferase increase.*

*^3^Drowsiness, anemia, nausea, bowel movement, increase energy, and headache.*

**FIGURE 2:**
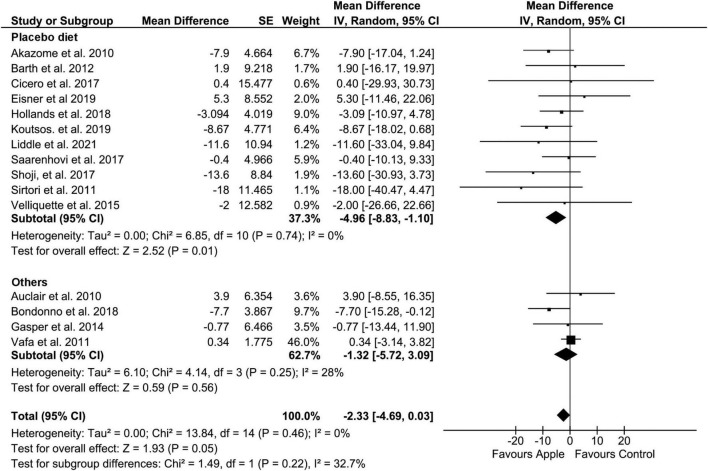
Meta-analysis results of total cholesterol. Forest plot is stratified by placebo diet or other alternative diets.

Overall, there was no statistically significant change in TC levels following apple and ADPs consumption in terms of blood concentration compared to those in the control group (MD: −2.33, 95% CI: −4.69, 0.03 mg/dL; *I*^2^ = 0%). However, a high tendency of reduction (*p* = 0.05) was observed (Figure 2). Notably, a significant reduction in TC levels was observed in the pooled analysis of 11 placebo-controlled trials (MD: −4.96, 95% CI: −8.83, −1.1 mg/dL; *I*^2^ = 0%). In addition, subgroup analysis of the study with a high baseline TC level also showed a significant reduction (MD: −3.26, 95% CI: −6.33, −0.19 mg/dL; *I*^2^ = 19%). In terms of administration forms, apple polyphenol was found to be likely to lower TC, while apple pectin (*n* = 2) significantly lowered TC in the treatment intervention subgroup analysis. Moreover, a significant reduction was observed when the sample type was confined to plasma (MD: −6.2, 95% CI: −11.29, −1.1, and *I*^2^ = 0%). All the outcomes of subgroup analysis and heterogeneity for TC, LDL, HDL, and TG are presented in Table 4.

**TABLE 4:** Meta-analysis summarization of total cholesterol (TC), low-density lipoprotein (LDL), high-density lipoprotein (HDL), triglycerides (TG), glucose, insulin, c-reactive protein (CRP), diastolic blood pressure (DBP), and systolic blood pressure (SBP).

Study group	Trials	Estimate effect		Heterogeneity			
		Weighted mean difference (95% CI)	*p*-effect	*Q* statistic	p-within group	*I*^2^%	*p*-between group
**Total cholesterol**							
Overall	15	−2.33 (−4.69, 0.03)	0.05	13.84	0.46	0	
Treatment intervention							0.23
Whole apple	4	−2.32 (−8.39, 3.75)	0.45	4.62	0.2	35	
Polyphenol	9	−3.48 (−7.14, 0.18)	0.06	4.42	0.82	0	
Apple pectin	2	−15.24 (−28.96, −1.52)	**0.03**	0.09	0.76	0	
Design							0.52
Crossover RCT	6	−3.91 (−7.74, −0.09)	0.05	4.24	0.51	0	
Parallel RCT	9	−2.15 (−5.92, 1.62)	0.26	8.53	0.38	6	
Sample type							0.09
Serum	9	−1.27 (−3.93, 1.39)	0.35	6.73	0.57	0	
Plasma	6	−6.2 (−11.29, −1.1)	**0.02**	4.29	0.51	0	
Basal condition							0.58
Healthy	2	−5.77 (−18.03, 6.49)	0.36	1.37	0.24	27	
CVD risk	13	−2.25 (−4.73, 0.24)	0.08	12.14	0.43	1	
Control intervention							0.22
Placebo	11	−4.96 (−8.83, −1.1)	**0.01**	6.85	0.74	0	
Others	4	−1.32 (−5.72, 3.09)	0.56	4.14	0.25	28	
Baseline level							0.71
<200 mg/dL	4	−1.09 (−11.96, 9.78)	0.84	1.5	0.68	0	
>200 mg/dL	11	−3.26 (−6.33, −0.19)	**0.04**	12.29	0.27	19	
**Low-density lipoproteins**							
Overall	16	−2.6 (−5.38, 0.19)	0.07	22.91	0.09	35	
Treatment intervention							0.2
Whole apple	5	−2.38 (−7.29, 2.53)	0.34	9.06	0.06	56	
Polyphenol	9	−2.22 (−5.46, 1.02)	0.18	7.9	0.44	0	
Apple pectin	2	−13.92 (−26.27, −1.57)	**0.03**	0	0.98	0	
Design							0.57
Crossover RCT	7	−2.74 (−6.65, 1.17)	0.17	9.91	0.13	39	
Parallel RCT	9	−1.27 (−4.56, 2.02)	0.45	8.96	0.35	11	
Sample type							0
Serum	9	0.64 (−1.16, 2.43)	0.49	7.59	0.47	0	
Plasma	7	−6.35 (−10.02, −2.68)	**<0.001**	4.06	0.67	0	
Basal condition							0.33
Healthy	3	−5.56 (−13.85, 2.73)	0.19	4.04	0.13	51	
CVD risk	13	−1.27 (−3.75, 1.22)	0.32	14.03	0.3	14	
Control intervention							0.35
Placebo	11	−4.03 (−7.39, −0.67)	**0.02**	4.08	0.94	0	
Others	5	−1.06 (−6.39, 4.27)	0.7	13.95	0.01	71	
Baseline level							0.24
<130 mg/dL	9	−0.98 (−3.73, 1.77)	0.48	9.37	0.31	15	
>130 mg/dL	7	−4.29 (−9.13, 0.55)	0.08	9.51	0.15	37	
**High-density lipoproteins**							
Overall	17	−1.01 (−1.71, −0.31)	**<0.001**	18.43	0.3	13	
Treatment intervention							0.85
Whole apple	6	−1.26 (−3.23, 0.72)	0.21	13.07	0.02	62	
Polyphenol	9	−0.6 (−1.61, 0.4)	0.24	3.92	0.86	0	
Apple pectin	2	−0.71 (−7.13, 5.7)	0.83	1.13	0.29	11	
Design							0.21
Crossover RCT	7	−1.84 (−3.54, −0.15)	**0.03**	12.61	0.05	52	
Parallel RCT	10	−0.74 (−1.15, −0.32)	**<0.001**	3.3	0.95	0	
Sample type							0.69
Serum	10	−0.8 (−1.22, −0.39)	**<0.001**	4.29	0.89	0	
Plasma	7	−1.25 (−3.34, 0.85)	0.24	13.46	0.04	55	
Basal condition							0.58
Healthy	4	−1.71 (−5.06, 1.65)	0.32	5.32	0.15	44	
CVD risk	13	−0.75 (−1.14, −0.35)	**<0.001**	5.75	0.93	0	
Control intervention							0.33
Placebo	11	−0.54 (−1.83, 0.76)	0.41	3.2	0.98	0	
Others	6	−1.53 (−3.04, −0.02)	**0.05**	14.96	0.01	67	
Baseline level							0.29
<50 mg/dL	6	−0.75 (−1.17, −0.33)	**<0.001**	1.84	0.87	0	
>50 mg/dL	11	−1.48 (−2.77, −0.19)	**0.02**	15.01	0.13	33	
**Triglycerides**							
Overall	17	−2.2 (−7.24, 2.85)	0.39	19.11	0.26	16	
Treatment intervention							0.93
Whole apple	6	−2.28 (−10.91, 6.35)	0.6	8.08	0.15	38	
Polyphenol	9	−1.99 (−9.87, 5.9)	0.62	10.84	0.21	26	
Apple pectin	2	2.34 (−20.41, 25.1)	0.84	0	0.96	0	
Design							0.2
Crossover RCT	7	−4.59 (−11.84, 2.65)	0.21	10.12	0.12	41	
Parallel RCT	10	2.24 (−5.28, 9.76)	0.56	6.55	0.68	0	
Sample type							0.44
Serum	10	−3.4 (−11.22, 4.42)	0.39	15.66	0.07	43	
Plasma	7	0.83 (−6.54, 8.2)	0.82	2.15	0.91	0	
Basal condition							0.84
Healthy	3	−1.01 (−10.69, 8.68)	0.84	0.46	0.79	0	
CVD risk	14	−2.18 (−8.52, 4.15)	0.5	18.52	0.14	30	
Control intervention							0.29
Placebo	10	−4.81 (−12.72, 3.1)	0.23	11.55	0.24	22	
Others	7	0.59 (−5.51, 6.68)	0.85	5.34	0.5	0	
Baseline level							0.02
<150 mg/dL	15	−4.37 (−9.05, 0.3)	0.07	13.46	0.49	0	
>150 mg/dL	2	13.2 (−0.74, 27.14)	0.06	0.17	0.68	0	
**Glucose**							
Overall	12	0.34 (−1.16, 1.84)	0.65	23.56	0.01	53	
**Insulin**							
Overall	9	−0.27 (−0.74, 0.21)	0.27	3.5	0.9	0	
**C-reactive protein**							
Overall	6	−0.03 (−0.34, 0.28)	0.84	17.42	0	71	
**Diastolic blood pressure**							
Overall	10	−0.34 (−1.52, 0.84)	0.57	10.93	0.28	18	
**Systolic blood pressure**							
Overall	10	−0.44 (−2.22, 1.33)	0.62	10.59	0.31	15	

*Subgroup analysis outcomes for glucose, insulin, C-reactive protein, SBP, and DBP are presented in Supplementary Material to compress the size of the current table. Bold values indicate the statistical significance.*

**FIGURE 3:**
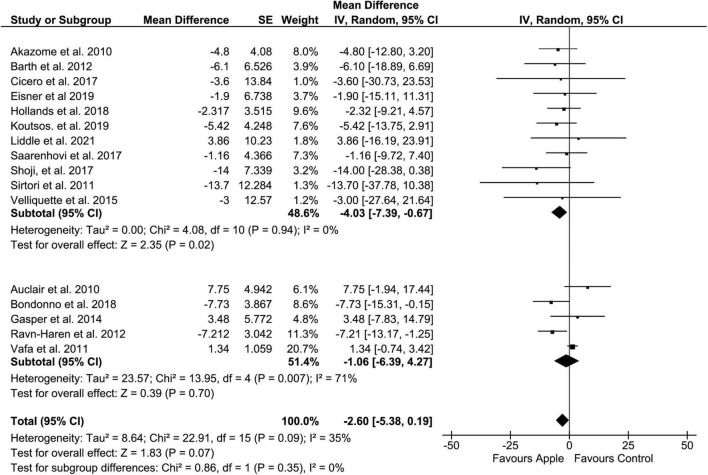
Meta-analysis results of low-density lipoprotein. Forest plot is stratified by placebo diet or other alternative diets.

Regarding LDL, when compared to the control diet, the overall result did not show a significant decrease in blood level (MD: −2.6, 95% CI: −5.38, 0.19 mg/dL; *I*^2^ = 35%). However, similar to TC, a clear tendency of reduction in LDL was easily recognized (*p* = 0.07) (Figure 3). Moreover, subgroup analysis on the placebo-controlled comparison showed a significant decrease in LDL (MD: −4.03, 95% CI: −7.39, −0.67 mg/dL; *I*^2^ = 0%). Regarding administration forms, apple pectin displayed a significant reduction in LDL concentration (MD: −13.92, 95% CI: −26.27, −1.57 mg/dL; *I*^2^ = 0%). The results were also similar to those of TC when the study was confined to the plasma sample (MD: −6.35, 95% CI: −10.02, −2.68 mg/dL, *I*^2^ = 0%).

**FIGURE 4:**
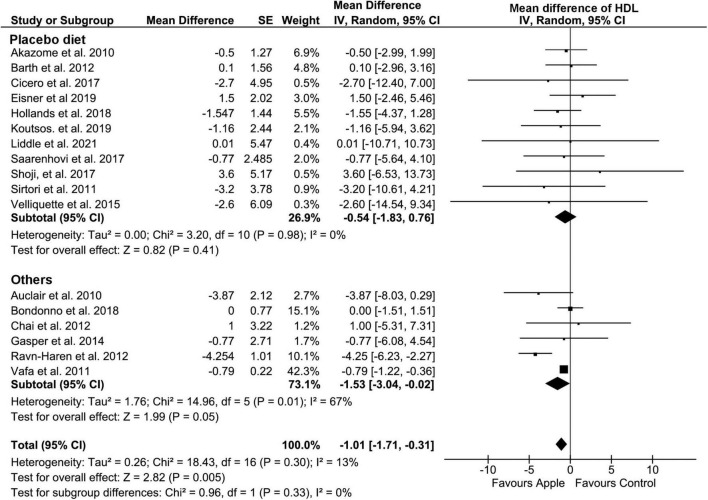
Meta-analysis results of high-density lipoprotein. Forest plot is stratified by placebo diet or other alternative diets.

**FIGURE 5:**
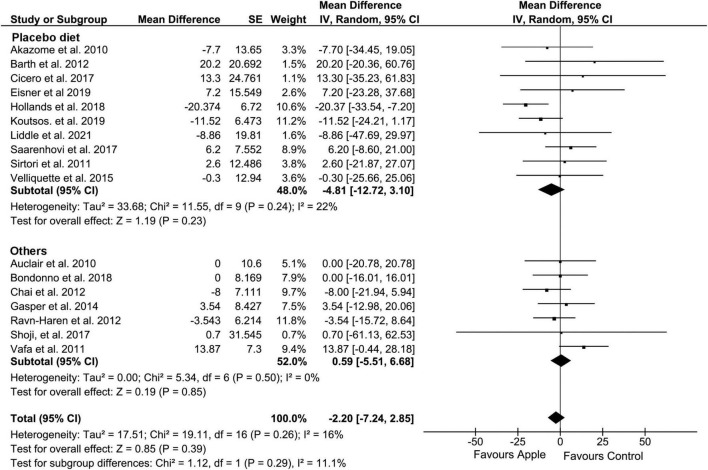
Meta-analysis results of triglycerides. Forest plot is stratified by placebo diet or other alternative diets.

Overall, HDL concentration was slightly but significantly decreased (MD: −1.01, 95% CI: −1.71, −0.31 mg/dL; *I*^2^ = 13%) (Figure 4). Most of the subgroup analyses also showed a slight reduction in HDL, except for the subgroup on the placebo-controlled comparison, healthy subjects, and the plasma sample. However, there was no evidence of alteration in TG compared to that in control diets (MD: −2.2, 95% CI: −7.24, 2.85 mg/dL; *I*^2^ = 16%) (Figure 5). Intriguingly, significant differences were observed when the subgroup was stratified to the high and low baseline TG value, as the subjects with low baseline (<150 mg/dL) showed a trend of reduction (MD: −4.37, 95% CI: −9.05, 0.3 mg/dL; *I*^2^ = 0%), whereas the opposites (>150 mg/dL) showed huge increase in the TG level (MD: 13.2, 95% CI: −0.74, 27.14 mg/dL; *I*^2^ = 0%).

### Glucose, Insulin, and C-Reactive Protein

Glucose, insulin, and CRP levels were reported in 12 studies (1, 19, 21, 23, 24, 26–28, 31, 33–35), nine studies (1, 19, 23, 24, 26–28, 32, 33), and six studies (20, 25, 27, 30, 34, 35), respectively. Serum and plasma samples were collected under fasting conditions in all included studies, except for the study conducted by Akazome et al. which did not provide clear information (23). No significant effects were found for glucose, insulin, or CRP levels. The results were consistent regardless of subgroup analysis. Details of the meta-analysis results are presented in Supplementary Figure 1 and Supplementary Table 3.

### Systolic Blood Pressure and Diastolic Blood Pressure

Alterations in SBP and DBP levels were reported in ten studies (1, 19, 21, 23, 28, 31–35). There were no significant changes in SBP and DBP in the overall outcomes, as well as in other subgroup analyses. The results of the overall meta-analysis and subgroup analysis are shown in Supplementary Figure 2 and Supplementary Table 4. Overall meta-analysis results of glucose, insulin, CRP, SBP, and DBP are presented in Table 4.

### Sensitivity Analysis

Sensitivity analysis revealed that several values that were close to the null hypothesis border became a significant result with a correlation coefficient of 0.8 and was conserved with an imputation of 0.2. The most notable alterations were the overall results of TC and LDL, which became significant with a correlation coefficient of 0.8 (MD: −3.71, 95% CI: −6.63, −0.79 mg/dL; *I*^2^ = 48%; MD: −2.98, 95% CI: −5.81, −0.15 mg/dL; *I*^2^ = 68%, respectively), whereas the non-significant values remained so when 0.2 was plugged in. Detailed results of the sensitivity analysis with different correlation coefficients are presented in Supplementary Tables 5, 6. Through leave-one-out sensitivity analysis, we found that the overall meta-analysis results of TC and LDL were highly influenced by a single study by Vafa et al. (22), because it accounted for 46.0 and 20.7% of the total weight, respectively, while it included only 23 participants in each group. Therefore, removing this study dramatically changed the overall outcomes of TC (MD: −4.60, 95% CI: −7.81, −1.39 mg/dL; *I*^2^ = 0%) and LDL (MD: −3.83, 95% CI: −6.39, −1.27 mg/dL; *I*^2^ = 0%) levels.

### Heterogeneity

*I*^2^ values for the overall meta-analysis ranged from 0% to 66%. We removed two studies (36, 37) because they were conducted in the same year and hospital with similar criteria, and showed inconsistent data reports. Therefore, the duplicated data was suspected. More importantly, a circumstance that the heterogeneity dramatically changed (e.g., from 0 to 92% for overall TC) in their sensitivity analysis, made us decide to exclude them from the meta-analysis. In addition, another study by Soriano-Maldonado et al. that investigated the effect of apple juice with different polyphenol and vitamin C concentrations was excluded after careful consideration due to the ambiguous study objective (38). As the study compared a mixture of two components with different concentrations, it was not feasible to indicate which is the treatment or control group as well as the component accounting for the main functionality for individual effects.

### Adverse Effects

From the 18 included trials, nine described information about the side effects potentially induced on account of the pharmacological use of apple (Table 3) (1, 19, 21, 23, 24, 29, 31, 33, 35). There were no observable adverse effects in six trials, whilst, non-fatal and mild side effects such as headache and rash were reported in 3 trials (24, 29, 35).

### Publication Bias

Tests of asymmetry showed a potential risk of publication bias on LDL (Egger’s test, *P* = 0.06) and SBP (Egger’s test, *P* = 0.01; Begg’s test, *P* = 0.09) (Supplementary Table 7). Nevertheless, no significant visual evidence of publication bias for LDL was observed when funnel plots were inspected (Supplementary Figure 3). There was no statistical evidence of publication bias for the other parameters measured by Egger’s regression test and Begg’s rank test.

### Quality Assessment

Individual quality assessment outcome is presented in Supplementary Table 8. The majority of included RCTs were evaluated to possess a certain risk of bias overall, mainly owing to the potential risk of bias due to deviations from the intended interventions. This was attributed to the general study design of the included RCTs, where they frequently treated apple products without concealing the flavor or smell, such as apple juice, or the whole apple itself.

### Quality of Evidence

The evidence profile of GRADE assessment is described in Supplementary Table 9. The confidence for reporting TC, LDL, TG, DBP, and SBP was rated as moderate quality (all downgraded for indirectness), whereas low quality for HDL, glucose, and insulin and very low quality for CRP were rated.

## Discussion

The current study aimed to introduce the present state of studies investigating the effect of apples on metabolic and cardiovascular markers. Consequently, 18 RCTs were systematically reviewed, and the commonly reported markers were meta-analyzed. Our study revealed significant reductions in TC and LDL concentrations upon apple and ADP intake when compared with the placebo control. Stratification by baseline level revealed potential beneficial effect of apple intake on the subjects with high TC and LDL. Intriguingly, HDL levels were also significantly decreased, although this cannot be considered beneficial. We found no evidence of changes in other markers, including TG, glucose, insulin, CRP, SBP, and DBP. To the best of our knowledge, this is the first comprehensive meta-analysis of RCTs regarding the effect of apples on metabolic and cardiovascular risk factors, which encompasses an unprecedentedly large sample size (up to 793 participants).

Although the current meta-analysis did not observe significant overall TC and LDL alteration, it revealed that consumption of apple and ADPs potentially lowers TC and LDL, especially in the subgroup with the so-called “placebo-controlled diet.” The absence of significant TC and LDL reduction in alterative diets potentially owes to other unidentified functional components. For example, the control intervention in the RCTs by Bondonno et al. and Auclair et al. was still a form of apple, albeit with a lower polyphenol concentration, and it may not rule out the effect of other components of the whole food matrix. Our findings broadly support previous systematic reviews and meta-analyses, which reported an inverse correlation between the intake of fruits and vegetables and the risk of CVD, including hypertension and coronary heart disease (CHD) (39–42). Gayer et al. conducted a meta-analysis on apples, pears, and their products and suggested the beneficial effects of apples and ADPs by reporting the trend of reduced risk of CVD and diabetes (10). Similarly, Launholt et al. conducted a systematic review of apple vinegar’s effects on metabolic parameters and suggested the potential blood glucose- and lipid-lowering effects of apple vinegar consumption (11).

The effects on TC and LDL were greater in the population with cardiovascular risk in the basal state. More specifically, a significant reduction in TC was observed in a group of people with high baseline cholesterol levels (>200 mg/dL), and a similar but non-significant trend was observed in the high LDL group (>130 mg/dL). Non-significant decreasing trends were also observed for TC and LDL when stratified for basal disease condition (CVD risk) indicated by each study, and removing data from Vafa et al. reversed the statistical outcome. In a recent meta-analysis of prospective studies, high apple and pear intakes were inversely associated with CHD and total stroke events (43). A prospective cohort study investigating the correlation between fruit intake and abdominal aortic calcification reported a significantly low odds ratio in a high apple intake fractile group (44). On the other hand, TG level was highly increased in high TG group (>150 mg/dL). However, it only included two research data and again, removing data from Vafa et al. turns the phenomenon back to baseline.

Even though there was an absence of statistical significance, the subgroup analysis related to treatment intervention suggested that the polyphenol-rich fraction of apples showed a high tendency to reduce TC and LDL. Both became significant after removal of the data from Auclair et al. The beneficial effects of apple and ADPs enriched with polyphenols have not been investigated in any other meta-analysis. The results of this study particularly revealed a direction for future research to explore the potential functions of apple polyphenols in lowering the risk of CVD. A possible explanation for the beneficial effects on the cardiovascular system is the abundance of phytochemicals such as procyanidins, hydroxycinnamic acids, and flavonols. These components have been investigated for their efficient antioxidant activity, lipid-lowering activity, and reduction in cardiotoxicity (45–48). Among the phenolic groups, anthocyanins were reported to have TC- and LDL-lowering effects, which were deduced by a meta-analysis of 30 and 27 RCTs (49). Dietary epicatechin, for which apples account for 28% of its intake, was also inversely correlated with CVD and CHD according to the 25-year follow-up data of elderly subjects (50). A recent longitudinal study on over 10,000 women with 12–15 years of follow-up reported a significantly low relative risk in the group with high flavone and flavonol intake, of which one of their major sources was apples (51). However, our analysis had limitations in that we could not separately consider all the various doses and ADP forms, which may be a potential reason for the absence of statistical significance. Therefore, additional well-designed RCTs on dose-response are warranted to elucidate the effect of apple polyphenols on lipid management. Apple pectin, a soluble fiber fraction, also significantly reduced in TC and LDL levels. However, the results should be interpreted with caution, as only a limited number of studies were involved in drawing this conclusion. Although the overall HDL level decreased with apple consumption, the quality of evidence was evaluated low according to GRADE assessment and more data are needed to confirm this phenomenon as a subgroup of placebo-controlled diets did not follow the overall outcome.

Overall, blood glucose, insulin, blood pressure, and CRP concentration were not associated with apple or ADP consumption. This could be explained by the fact that most of the included studies investigated these factors as secondary outcomes. A few studies have investigated the attributes of apple polyphenols in glycemic management, and most of them focused on acute post-prandial responses (52–54). However, apples have been suggested to improve insulin sensitivity (55, 56). Notably, subgroup analysis of serum sample type revealed a potential effect of apple intake on the insulin sensitivity increase (MD: −0.51, 95% CI: −1.07, 0.05 μU/mL; *p* = 0.07; *I*^2^ = 0%). Considering the general consensus that serum is recommended over plasma for blood insulin measurement (57), we expect further trials to reverse the current outcome. Despite the lack of significant favorable effects observed in our present meta-analysis, several studies have reported the beneficial effects of apple extract on blood pressure and endothelial function (31, 58). However, the evidence remains uncertain due to the inconsistency of results presented in our meta-analysis. Finally, the effect of apples and ADPs on CRP showed no potential association except in a subgroup analysis of two studies on plasma samples.

There are a few limitations to this study that need to be addressed. First, some subgroups (e.g., apple pectin) have a limited number of studies; therefore, the statistical power is relatively weak for such an analysis. A dose-dependent analysis was not conducted in our study, neither was a more sub-categorized analysis of each specific type of apple intervention. This is because various specific components were used in each study, for example, flavanol, epicatechin, or total polyphenol in the apple polyphenol group. In the same vein, the heterogeneity in the intervention of apple and control positions all 9 markers at below the good quality of evidence. Finally, there have been multiple data imputations due to missing correlation information, which potentially distorts the original outcome. To avoid bias due to imputation, we counter-checked the results with a sensitivity analysis by plugging in different correlation coefficients including 0.2 and 0.8.

## Conclusion

The current meta-analysis demonstrated that more than a week of apple and ADP intake can reduce TC and LDL levels when compared to a placebo-controlled diet. HDL levels were also significantly reduced upon apple consumption, while TG, glucose, insulin, CRP, SBP, and DBP were unaffected (*p* > 0.05) by the intervention. We anticipate that our results will guide the direction of future investigations and be statistically reinforced by further follow-up clinical trials with larger populations and longer study periods.

## Data Availability Statement

The original contributions presented in the study are included in the article/Supplementary Material, further inquiries can be directed to the corresponding author/s.

## Author Contributions

SJK, NA, NL, and SWK designed the research (project conception, development of overall research plan, and study oversight). SJK, NA, and CWJ conducted the research (systematic search, article screening, and data synthesis). NA, YCY, EGL, MK, and EYS conducted the research (data extraction). SJK, CWJ, THK, and YD conducted the research (quality assessment). SP, YHC, and JL performed the statistical analysis. SJK and NA wrote the manuscript. SWK had primary responsibility for final content. All authors read and approved the final manuscript.

## Conflict of Interest

The authors declare that the research was conducted in the absence of any commercial or financial relationships that could be construed as a potential conflict of interest.

## Publisher’s Note

All claims expressed in this article are solely those of the authors and do not necessarily represent those of their affiliated organizations, or those of the publisher, the editors and the reviewers. Any product that may be evaluated in this article, or claim that may be made by its manufacturer, is not guaranteed or endorsed by the publisher.
