# Supplementary material for: Metabolic and Cardiovascular Benefits of Apple and Apple-Derived Products: A Systematic Review and Meta-Analysis of Randomized Controlled Trials
Source: Front Nutr. 2022 Apr 5;9:766155. doi: 10.3389/fnut.2022.766155 (PMC9016272; doi:10.3389/fnut.2022.766155)
Supplement: Supplementary file 1 [file Data_Sheet_1.docx]

Supplementary Material

# Supplementary Figures and Tables

## Supplementary Figures


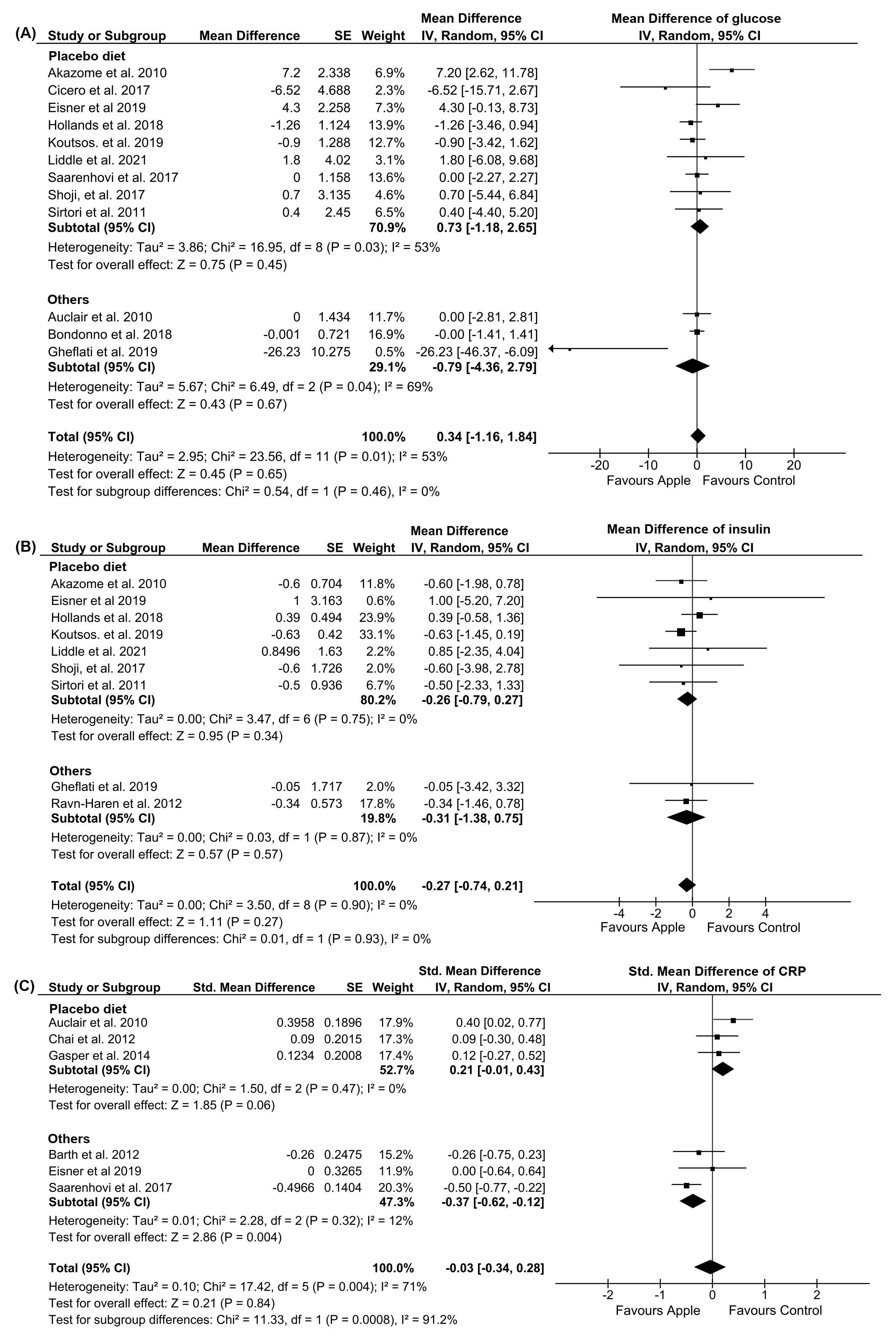
**Supplementary Figure 1.** Forest plot of control intervention subtype: **(A)** Glucose, (**B)** Insulin, (**C)** C-reactive protein (CRP).


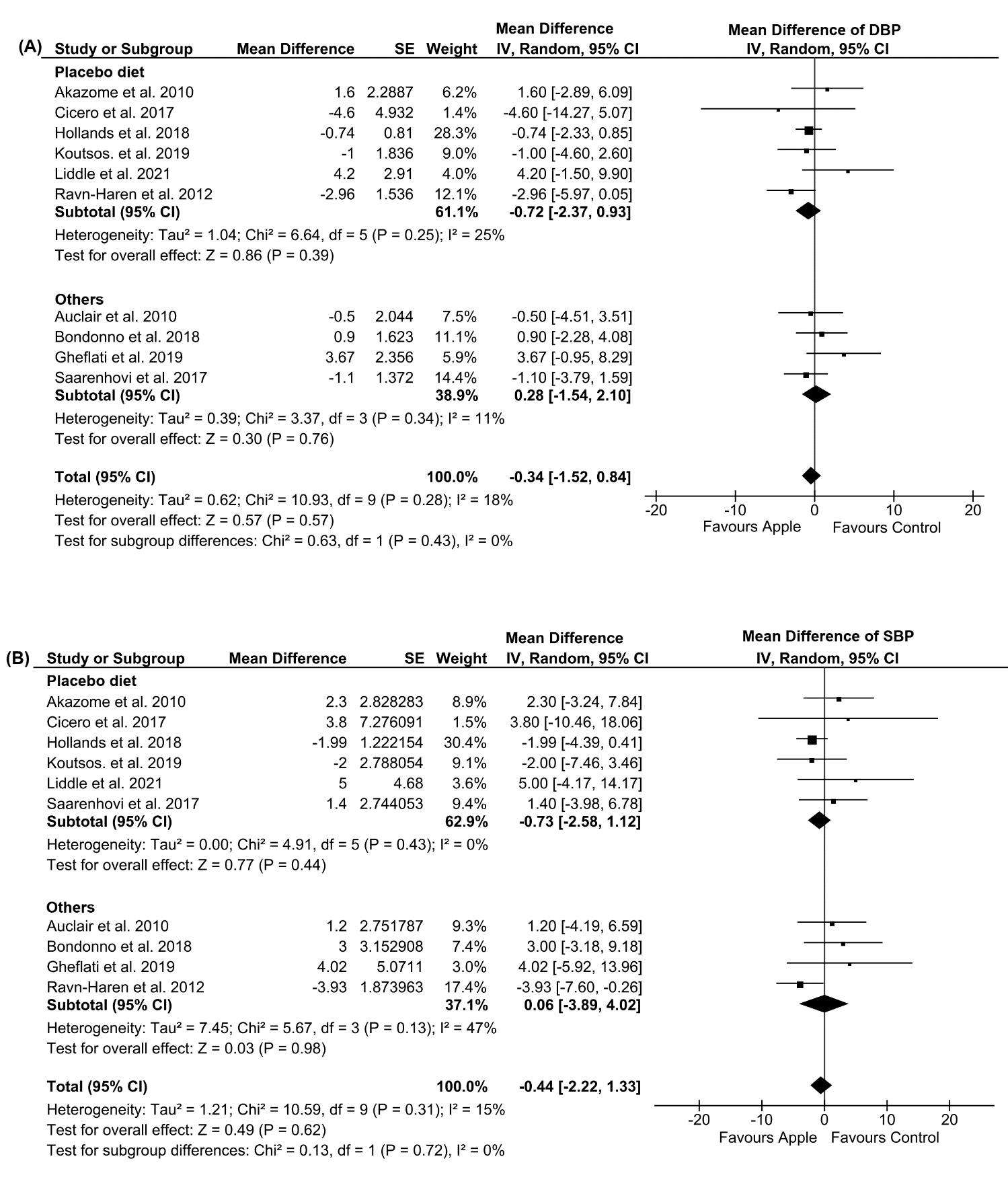


**Supplementary Figure 2.** Forest plot of control intervention subtype: (**A**) Diastolic blood pressure (DBP), (**B**) Systolic blood pressure (SBP).


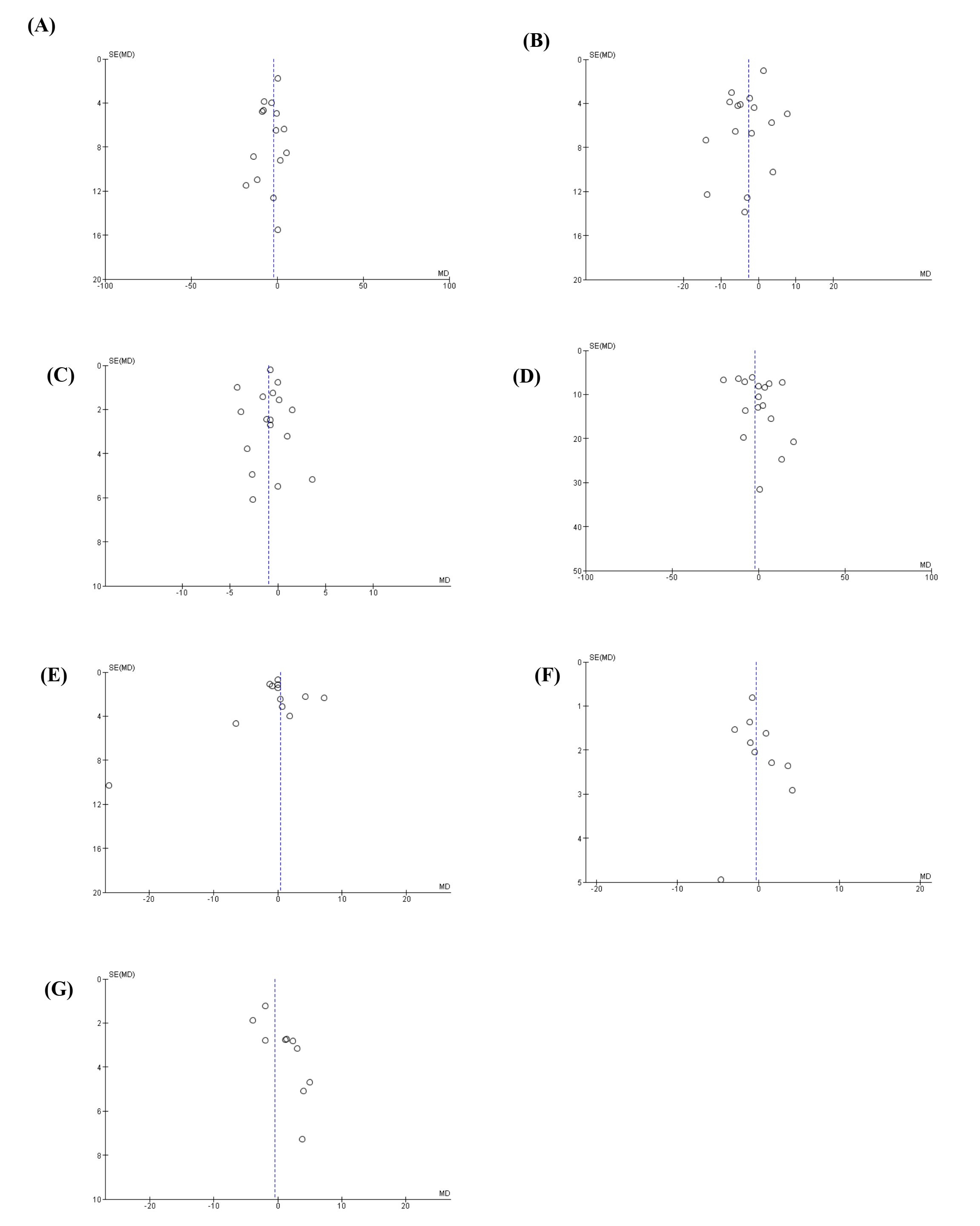
**Supplementary Figure 3.** Funnel plot of meta-analysis result: **(A)** Total cholesterol, (**B)** Low-density lipoprotein, (**C)** High-density lipoprotein, (**D)** Triglycerides, (**E)** Glucose, (**F**) Diastolic blood pressure, (**G**) Systolic blood pressure

## Supplementary Tables

**Supplementary Table 1.** PRISMA checklist 2020.

| **Section and Topic** | **Item #** | **Checklist item** | **Location where item is reported** |
| --- | --- | --- | --- |
| **TITLE** | | | |
| Title | 1 | Identify the report as a systematic review. | Page 1 |
| **ABSTRACT** | | | |
| Abstract | 2 | See the PRISMA 2020 for Abstracts checklist. | Page 2 |
| **INTRODUCTION** | | | |
| Rationale | 3 | Describe the rationale for the review in the context of existing knowledge. | Page 3 |
| Objectives | 4 | Provide an explicit statement of the objective(s) or question(s) the review addresses. | Page 3 |
| **METHODS** | | | |
| Eligibility criteria | 5 | Specify the inclusion and exclusion criteria for the review and how studies were grouped for the syntheses. | Pages 3-4 |
| Information sources | 6 | Specify all databases, registers, websites, organisations, reference lists and other sources searched or consulted to identify studies. Specify the date when each source was last searched or consulted. | Page 3 |
| Search strategy | 7 | Present the full search strategies for all databases, registers and websites, including any filters and limits used. | Page 3 , Supplementary Table 2 |
| Selection process | 8 | Specify the methods used to decide whether a study met the inclusion criteria of the review, including how many reviewers screened each record and each report retrieved, whether they worked independently, and if applicable, details of automation tools used in the process. | Pages 3-4 |
| Data collection process | 9 | Specify the methods used to collect data from reports, including how many reviewers collected data from each report, whether they worked independently, any processes for obtaining or confirming data from study investigators, and if applicable, details of automation tools used in the process. | Page 4 |
| Data items | 10a | List and define all outcomes for which data were sought. Specify whether all results that were compatible with each outcome domain in each study were sought (e.g. for all measures, time points, analyses), and if not, the methods used to decide which results to collect. | Pages 4-5 |
|  | 10b | List and define all other variables for which data were sought (e.g. participant and intervention characteristics, funding sources). Describe any assumptions made about any missing or unclear information. | Pages 4-5 |
| Study risk of bias assessment | 11 | Specify the methods used to assess risk of bias in the included studies, including details of the tool(s) used, how many reviewers assessed each study and whether they worked independently, and if applicable, details of automation tools used in the process. | Page 5-6 |
| Effect measures | 12 | Specify for each outcome the effect measure(s) (e.g. risk ratio, mean difference) used in the synthesis or presentation of results. | Pages 4-5 |
| Synthesis methods | 13a | Describe the processes used to decide which studies were eligible for each synthesis (e.g. tabulating the study intervention characteristics and comparing against the planned groups for each synthesis (item #5)). | Page 4-5 |
|  | 13b | Describe any methods required to prepare the data for presentation or synthesis, such as handling of missing summary statistics, or data conversions. | Pages 4-5 , Table 1, 2 |
|  | 13c | Describe any methods used to tabulate or visually display results of individual studies and syntheses. | Pages 4-5 |
|  | 13d | Describe any methods used to synthesize results and provide a rationale for the choice(s). If meta-analysis was performed, describe the model(s), method(s) to identify the presence and extent of statistical heterogeneity, and software package(s) used. | Pages 4-5 |
|  | 13e | Describe any methods used to explore possible causes of heterogeneity among study results (e.g. subgroup analysis, meta-regression). | Pages 5 |
|  | 13f | Describe any sensitivity analyses conducted to assess robustness of the synthesized results. | Page 5 |
| Reporting bias assessment | 14 | Describe any methods used to assess risk of bias due to missing results in a synthesis (arising from reporting biases). | Page 5 |
| Certainty assessment | 15 | Describe any methods used to assess certainty (or confidence) in the body of evidence for an outcome. | Page 6 |
| **RESULTS** | | | |
| Study selection | 16a | Describe the results of the search and selection process, from the number of records identified in the search to the number of studies included in the review, ideally using a flow diagram. | Page 6, Figure 1 |
|  | 16b | Cite studies that might appear to meet the inclusion criteria, but which were excluded, and explain why they were excluded. | Page 6, Figure 1 |
| Study characteristics | 17 | Cite each included study and present its characteristics. | Page 6, Table 3 |
| Risk of bias in studies | 18 | Present assessments of risk of bias for each included study. | Page 9 , Supplementary Table 8 |
| Results of individual studies | 19 | For all outcomes, present, for each study: (a) summary statistics for each group (where appropriate) and (b) an effect estimate and its precision (e.g. confidence/credible interval), ideally using structured tables or plots. | Pages 6-9 |
| Results of syntheses | 20a | For each synthesis, briefly summarise the characteristics and risk of bias among contributing studies. | Pages 7-8 |
|  | 20b | Present results of all statistical syntheses conducted. If meta-analysis was done, present for each the summary estimate and its precision (e.g. confidence/credible interval) and measures of statistical heterogeneity. If comparing groups, describe the direction of the effect. | Pages 7-8 , Table 4, Supplementary Tables 3 and 4 |
|  | 20c | Present results of all investigations of possible causes of heterogeneity among study results. | Pages 8, Supplementary Table 7 |
|  | 20d | Present results of all sensitivity analyses conducted to assess the robustness of the synthesized results. | Page 8, Supplementary Tables 5 and 6 |
| Reporting biases | 21 | Present assessments of risk of bias due to missing results (arising from reporting biases) for each synthesis assessed. | Page 8, Supplementary Figure 3, Supplementary Table 7 |
| Certainty of evidence | 22 | Present assessments of certainty (or confidence) in the body of evidence for each outcome assessed. | Page 9, Supplementary Table 9 |
| **DISCUSSION** | | | |
| Discussion | 23a | Provide a general interpretation of the results in the context of other evidence. | Pages 9-11 |
|  | 23b | Discuss any limitations of the evidence included in the review. | Pages 9-11 |
|  | 23c | Discuss any limitations of the review processes used. | Pages 9-11 |
|  | 23d | Discuss implications of the results for practice, policy, and future research. | Page 11 |
| **OTHER INFORMATION** | | | |
| Registration and protocol | 24a | Provide registration information for the review, including register name and registration number, or state that the review was not registered. | Page 2 |
|  | 24b | Indicate where the review protocol can be accessed, or state that a protocol was not prepared. | Page 2 |
|  | 24c | Describe and explain any amendments to information provided at registration or in the protocol. | NA |
| Support | 25 | Describe sources of financial or non-financial support for the review, and the role of the funders or sponsors in the review. | Page 11 |
| Competing interests | 26 | Declare any competing interests of review authors. | Page11 |
| Availability of data, code and other materials | 27 | Report which of the following are publicly available and where they can be found: template data collection forms; data extracted from included studies; data used for all analyses; analytic code; any other materials used in the review. | NA |

**Supplementary Table 2.** Searching query of four databases.

| **Database name** | **Query** |
| --- | --- |
| **PUBMED** | #1: (apple OR apples OR “Malus domestica” OR “M. domestica” OR “Malus pumila Mill.” OR “Malus pumila” OR “M. pumila”)  #2: (“lipid profile” OR “lipid distribution” OR lipid OR cholesterol OR “total cholesterol” OR TC OR “low density lipoprotein” OR LDL OR “LDL-C” OR “high density lipoprotein” OR HDL OR “HDL-C” OR triglyceride OR triacylglycerol OR TG OR TAG OR lipoprotein OR CRP OR “C-reactive protein” OR “blood pressure” OR “systolic blood pressure” OR SBP OR “diastolic blood pressure” OR “DBP” OR “insulin” OR “cardiovascular” OR “cardiovascular disease” OR “CVD” OR “diabetes” OR “glucose” OR “blood glucose” OR “fasting glucose” OR “glycemic”)  #3 ("randomized controlled trial"[pt] OR "controlled clinical trial"[pt] OR "randomized"[tiab] OR "placebo"[tiab] OR "drug therapy"[mh] OR "randomly"[tiab] OR "trial"[tiab] OR "groups"[tiab]) NOT ("animals"[mh] NOT "humans"[mh])  #4: #1 AND #2  #5: #3 AND #4 |
| **EMABSE** | #1: (apple OR apples OR 'malus domestica' OR 'm. domestica' OR 'malus pumila mill.' OR 'malus pumila' OR 'm. pumila')  #2: ('lipid profile' OR 'lipid distribution' OR lipid OR cholesterol OR 'total cholesterol' OR tc OR 'low density lipoprotein' OR ldl OR 'ldl-c' OR 'high density lipoprotein' OR hdl OR 'hdl-c' OR triglyceride OR triacylglycerol OR tg OR tag OR lipoprotein OR crp OR 'c-reactive protein' OR 'blood pressure' OR 'systolic blood pressure' OR sbp OR 'diastolic blood pressure' OR 'dbp' OR 'insulin' OR 'cardiovascular' OR 'cardiovascular disease' OR 'cvd' OR 'diabetes' OR 'glucose' OR 'blood glucose' OR 'fasting glucose' OR 'glycemic')  #3: ('intervention studies'/exp OR 'randomized':ab OR 'randomised':ab OR 'randomly':ab OR 'random':ab OR 'controlled trial':ab OR 'interventions':ab OR 'placebo':ab OR 'assignment':ab) AND [embase]/lim  #4: #1 AND #2  #5: #3 AND #4 |
| **CENTRAL** | #1: (apple OR apples OR “Malus domestica” OR “M. domestica” OR “Malus pumila Mill.” OR “Malus pumila” OR “M. pumila”)  #2: (“lipid profile” OR “lipid distribution” OR lipid OR cholesterol OR “total cholesterol” OR TC OR “low density lipoprotein” OR LDL OR “LDL-C” OR “high density lipoprotein” OR HDL OR “HDL-C” OR triglyceride OR triacylglycerol OR TG OR TAG OR lipoprotein OR CRP OR “C-reactive protein” OR “blood pressure” OR “systolic blood pressure” OR SBP OR “diastolic blood pressure” OR “DBP” OR “insulin” OR “cardiovascular” OR “cardiovascular disease” OR “CVD” OR “diabetes” OR “glucose” OR “blood glucose” OR “fasting glucose” OR “glycemic”)  #3: #1 AND #2 |
| **ClinicalTrials.gov** | #1: (apple OR apples OR “Malus domestica” OR “M. domestica” OR “Malus pumila Mill.” OR “Malus pumila” OR “M. pumila”) |

**Supplementary Table 3.** Meta-analysis summarization of glucose, insulin, and C-reactive protein.

| **Study group** | **Trials** | **Estimate effect** | | **Heterogeneity** | | |  |
| --- | --- | --- | --- | --- | --- | --- | --- |
|  |  | **Weighted mean difference (95% CI)** | **p-effect^*^** | **Q statistic** | **p-within group** | **I^2^%** | **p-between group** |
| **Glucose** | | | | | | | |
| Overall | 12 | 0.34 (-1.16, 1.84) | 0.65 | 23.56 | 0.01 | 53 |  |
| Treatment intervention |  |  |  |  |  |  | 0.08 |
| Whole apple | 3 | 1.29 (-2.4, 4.98) | 0.49 | 4.12 | 0.13 | 51 |  |
| Polyphenol | 6 | 0.25 (-1.53, 2.03) | 0.78 | 12.67 | 0.03 | 61 |  |
| Apple pectin | 2 | 0.51 (-3.27, 4.3) | 0.79 | 0.01 | 0.94 | 0 |  |
| Apple vinegar | 1 | -26.23 (-46.37, -6.09) | **0.01** | NA | NA | NA |  |
| Design |  |  |  |  |  |  | 0.71 |
| Crossover RCT | 5 | -0.34 (-1.26, 0.58) | 0.47 | 1.22 | 0.87 | 0 |  |
| Parallel RCT | 7 | 0.92 (-3.24, 5.08) | 0.66 | 17.29 | 0.01 | 65 |  |
| Sample type |  |  |  |  |  |  | 0.59 |
| Serum | 6 | 0.85 (-2.22, 3.91) | 0.59 | 21.31 | 0 | 77 |  |
| Plasma | 6 | -0.02 (-1.14, 1.11) | 0.98 | 2.21 | 0.82 | 0 |  |
| Basal condition |  |  |  |  |  |  | 0.9 |
| Healthy | 1 | 0.7 (-5.44, 6.84) | 0.82 | NA | NA | NA |  |
| CVD risk | 11 | 0.33 (-1.25, 1.92) | 0.68 | 23.52 | 0.01 | 57 |  |
| Control intervention |  |  |  |  |  |  | 0.48 |
| Placebo | 9 | 0.73 (-1.18, 2.65) | 0.45 | 16.95 | 0.03 | 53 |  |
| Others | 3 | -0.79 (-4.36, 2.79) | 0.67 | 6.49 | 0.04 | 69 |  |
| Baseline level |  |  |  |  |  |  | 0.01 |
| <130 mg/dL | 11 | 0.38 (-0.91, 1.67) | 0.56 | 17 | 0.07 | 41 |  |
| >130 mg/dL | 1 | -26.23 (-46.37, -6.09) | **0.01** | NA | NA | NA |  |
| **Insulin** | | | | | | | |
| Overall | 9 | -0.27 (-0.74, 0.21) | 0.27 | 3.5 | 0.9 | 0 |  |
| Treatment intervention |  |  |  |  |  |  | 0.86 |
| Whole apple | 4 | -0.46 (-1.1, 0.19) | 0.17 | 1.07 | 0.79 | 0 |  |
| Polyphenol | 2 | 0.02 (-0.92, 0.96) | 0.96 | 1.33 | 0.25 | 25 |  |
| Apple pectin | 2 | -0.52 (-2.14, 1.09) | 0.53 | 0 | 0.96 | 0 |  |
| Apple vinegar | 1 | -0.05 (-3.42, 3.32) | 0.98 | NA | NA | NA |  |
| Design |  |  |  |  |  |  | 0.8 |
| Crossover RCT | 3 | -0.22 (-0.84, 0.4) | 0.48 | 2.52 | 0.28 | 21 |  |
| Parallel RCT | 6 | -0.37 (-1.31, 0.58) | 0.45 | 0.93 | 0.97 | 0 |  |
| Sample type |  |  |  |  |  |  | 0.11 |
| Serum | 6 | -0.51 (-1.07, 0.05) | 0.07 | 0.49 | 0.99 | 0 |  |
| Plasma | 3 | 0.36 (-0.54, 1.25) | 0.43 | 0.4 | 0.82 | 0 |  |
| Basal condition |  |  |  |  |  |  | 0.84 |
| Healthy | 2 | -0.37 (-1.43, 0.7) | 0.5 | 0.02 | 0.89 | 0 |  |
| CVD risk | 7 | -0.24 (-0.77, 0.28) | 0.37 | 3.44 | 0.75 | 0 |  |
| Control intervention |  |  |  |  |  |  | 0.93 |
| Placebo | 7 | -0.26 (-0.79, 0.27) | 0.34 | 3.47 | 0.75 | 0 |  |
| Others | 2 | -0.31 (-1.38, 0.75) | 0.57 | 0.03 | 0.87 | 0 |  |
| **C-reactive protein** | | | | | | | |
| Overall | 6 | -0.03 (-0.34, 0.28) | 0.84 | 17.42 | 0 | 71 |  |
| Treatment intervention |  |  |  |  |  |  | 0.52 |
| Whole apple | 2 | 0.07 (-0.27, 0.4) | 0.7 | 0.06 | 0.81 | 0 |  |
| Polyphenol | 4 | -0.06 (-0.5, 0.37) | 0.77 | 16.38 | 0 | 82 |  |
| Design |  |  |  |  |  |  | 0.65 |
| Crossover RCT | 3 | 0 (-0.57, 0.56) | 0.99 | 16.05 | 0 | 87 |  |
| Parallel RCT | 3 | -0.04 (-0.32, 0.24) | 0.78 | 1.22 | 0.54 | 0 |  |
| Sample type |  |  |  |  |  |  | <0.001 |
| Serum | 4 | 0.19 (-0.02, 0.4) | 0.08 | 1.87 | 0.6 | 0 |  |
| Plasma | 2 | -0.44 (-0.68, -0.2) | **<0.001** | 0.69 | 0.41 | 0 |  |
| Basal condition |  |  |  |  |  |  | 0.44 |
| Healthy | 1 | 0.12 (-0.27, 0.52) | 0.54 | NA | NA | NA |  |
| CVD risk | 5 | -0.06 (-0.43, 0.3) | 0.74 | 16.13 | 0 | 75 |  |
| Control intervention |  |  |  |  |  |  | 0.13 |
| Placebo | 3 | 0.21 (-0.01, 0.43) | 0.06 | 1.5 | 0.47 | 0 |  |
| Others | 3 | -0.37 (-0.62, -0.12) | **<0.001** | 2.28 | 0.32 | 12 |  |
| Baseline level |  |  |  |  |  |  | 0.64 |
| <10 mg/dL | 5 | -0.06 (-0.43, 0.32) | 0.77 | 16.52 | 0 | 75.79 |  |
| >10 mg/dL | 1 | 0.09 (-0.3, 0.48) | 0.66 | NA | NA | NA |  |
| **^*^** Bold value indicates the statistical significance. | | | | | | | |

**Supplementary Table 4.** Meta-analysis summarization of diastolic and systolic blood pressure

| **Study group** | **Trials** | **Estimate effect** | | **Heterogeneity** | | | |  | |
| --- | --- | --- | --- | --- | --- | --- | --- | --- | --- |
|  |  | **Weighted mean difference (95% CI)** | **p-effect^*^** | **Q statistic** | **p-within group** | **I^2^%** | **p-between group** | | |
| **Diastolic blood pressure** | | | | | | | | |  |
| Overall | 10 | -0.34 (-1.52, 0.84) | 0.57 | 10.93 | 0.28 | 18 |  | | |
| Treatment intervention |  |  |  |  |  |  | 0.23 | | |
| Whole apple | 3 | -0.61 (-4.12, 2.91) | 0.74 | 4.77 | 0.09 | 58 |  | | |
| Polyphenol | 6 | -0.47 (-1.62, 0.68) | 0.42 | 2.55 | 0.77 | 0 |  | | |
| Apple vinegar | 1 | 3.67 (-0.95, 8.29) | 0.12 | NA | NA | NA |  | | |
| Design |  |  |  |  |  |  | 0.03 | | |
| Crossover RCT | 6 | -0.9 (-1.96, 0.17) | 0.1 | 3.13 | 0.68 | 0 |  | | |
| Parallel RCT | 4 | 2.4 (-0.29, 5.09) | 0.08 | 2.81 | 0.42 | 0 |  | | |
| Basal condition |  |  |  |  |  |  | 0.08 | | |
| Healthy | 9 | -0.15 (-1.19, 0.9) | 0.78 | 7.93 | 0.44 | 0 |  | | |
| CVD risk | 1 | -2.96 (-5.97, 0.05) | 0.05 | NA | NA | NA |  | | |
| Control intervention |  |  |  |  |  |  | 0.43 | | |
| Placebo | 6 | -0.72 (-2.37, 0.93) | 0.39 | 6.64 | 0.25 | 25 |  | | |
| Others | 4 | 0.28 (-1.54, 2.1) | 0.76 | 3.37 | 0.34 | 11 |  | | |
| Baseline level |  |  |  |  |  |  | 0.55 | | |
| <80 mmHg | 8 | -0.54 (-1.74, 0.66) | 0.38 | 7.6 | 0.37 | 8 |  | | |
| >80 mmHg | 2 | 0.9 (-3.71, 5.51) | 0.7 | 3.06 | 0.08 | 67 |  | | |
| **Systolic blood pressure** | | | | | | | | |  |
| Overall | 10 | -0.44 (-2.22, 1.33) | 0.62 | 10.59 | 0.31 | 15 |  | | |
| Treatment intervention |  |  |  |  |  |  | 0.53 | | |
| Whole apple | 3 | -1.87 (-5.91, 2.17) | 0.37 | 3.18 | 0.2 | 37 |  | | |
| Polyphenol | 6 | -0.28 (-2.08, 1.53) | 0.76 | 4.85 | 0.43 | 0 |  | | |
| Apple vinegar | 1 | 4.02 (-5.92, 13.96) | 0.43 | NA | NA | NA |  | | |
| Design |  |  |  |  |  |  | 0.05 | | |
| Crossover RCT | 6 | -1.25 (-3.14, 0.64) | 0.19 | 5.98 | 0.31 | 16 |  | | |
| Parallel RCT | 4 | 3.26 (-0.84, 7.36) | 0.12 | 0.28 | 0.96 | 0 |  | | |
| Basal condition |  |  |  |  |  |  | 0.07 | | |
| Healthy | 1 | -3.93 (-7.6, -0.26) | **0.04** | NA | NA | NA |  | | |
| CVD risk | 9 | -0.14 (-1.8, 1.52) | 0.87 | 7.2 | 0.52 | 0 |  | | |
| Control intervention |  |  |  |  |  |  | 0.72 | | |
| Placebo | 6 | -0.73 (-2.58, 1.12) | 0.44 | 4.91 | 0.43 | 0 |  | | |
| Others | 4 | 0.06 (-3.89, 4.02) | 0.98 | 5.67 | 0.13 | 47 |  | | |
| Baseline level |  |  |  |  |  |  | 0.24 | | |
| >120 mmHg | 8 | -0.84 (-2.74, 1.07) | 0.39 | 8.35 | 0.3 | 16 |  | | |
| <120 mmHg | 2 | 2.18 (-2.47, 6.83) | 0.36 | 0.49 | 0.48 | 0 |  | | |
| **^*^** Bold value indicates the statistical significance. | | | | | | | | | |

**Supplementary Table 5.** Sensitivity analysis with correlation imputation of 0.8.

| **Study group** | **Trials** | **Estimate effect** | | **Heterogeneity** | | | | |  |
| --- | --- | --- | --- | --- | --- | --- | --- | --- | --- |
|  |  | **Weighted mean difference (95% CI)** | **p-effect^*^** | **Q statistic** | **p-within group** | **I^2^%** | **p-between group** | |  |
| **Total cholesterol** | | | | | | | |  |  |
| Overall | 15 | -3.71 (-6.63, -0.79) | **0.01** | 27.17 | 0.02 | 48 |  | |  |
| Treatment intervention |  |  |  |  |  |  | 0.03 | |  |
| Whole apple | 4 | -3.45 (-10.23, 3.33) | 0.32 | 9.85 | 0.02 | 70 |  | |  |
| Polyphenol | 9 | -2.8 (-5.62, 0.03) | 0.05 | 9.47 | 0.3 | 16 |  | |  |
| Apple pectin | 2 | -15.3 (-24.29, -6.31) | **<0.001** | 0.22 | 0.64 | 0 |  | |  |
| Design |  |  |  |  |  |  | 0.69 | |  |
| Crossover RCT | 6 | -3.38 (-7.04, 0.28) | 0.07 | 10.57 | 0.06 | 53 |  | |  |
| Parallel RCT | 9 | -4.65 (-9.81, 0.51) | 0.08 | 15.66 | 0.05 | 49 |  | |  |
| Sample type |  |  |  |  |  |  | 0.12 | |  |
| Serum | 9 | -1.99 (-5.09, 1.1) | 0.21 | 11.91 | 0.16 | 33 |  | |  |
| Plasma | 6 | -6.89 (-12.28, -1.50) | **0.01** | 10.39 | 0.06 | 52 |  | |  |
| Basal condition |  |  |  |  |  |  | 0.64 | |  |
| Healthy | 2 | -6.5 (-19.01, 6) | 0.31 | 3.22 | 0.07 | 69 |  | |  |
| CVD risk | 13 | -3.43 (-6.52, -0.34) | **0.03** | 23.62 | 0.02 | 49 |  | |  |
| Control intervention |  |  |  |  |  |  | 0.24 | |  |
| Placebo | 11 | -5.12 (-8.72, -1.53) | **0.01** | 14.88 | 0.14 | 33 |  | |  |
| Others | 4 | -1.47 (-6.44, 3.51) | 0.56 | 9.33 | 0.03 | 68 |  | |  |
| Baseline level |  |  |  |  |  |  | 0.7 | |  |
| <200 mg/dL | 4 | -2.21 (-10.31, 5.89) | 0.59 | 3.25 | 0.35 | 8 |  | |  |
| >200 mg/dL | 11 | -3.94 (-7.15, -0.72) | **0.02** | 23.85 | 0.01 | 58 |  | |  |
| **Low-density lipoproteins** | | | | | | | |  |  |
| Overall | 16 | -2.98 (-5.81, -0.15) | **0.04** | 46.93 | 0 | 68 |  | |  |
| Treatment intervention |  |  |  |  |  |  | 0.02 | |  |
| Whole apple | 5 | -2.62 (-7.5, 2.26) | 0.29 | 18.48 | 0 | 78 |  | |  |
| Polyphenol | 9 | -1.69 (-5.37, 2) | 0.37 | 18.93 | 0.02 | 58 |  | |  |
| Apple pectin | 2 | -13.92 (-21.91, -5.93) | **<0.001** | 0 | 0.97 | 0 |  | |  |
| Design |  |  |  |  |  |  | 0.55 | |  |
| Crossover RCT | 7 | -2.23 (-6.14, 1.69) | 0.27 | 24.55 | 0 | 76 |  | |  |
| Parallel RCT | 9 | -4.04 (-8.56, 0.48) | 0.08 | 17.38 | 0.03 | 54 |  | |  |
| Sample type |  |  |  |  |  |  | 0.01 | |  |
| Serum | 9 | -0.24 (-3.14, 2.66) | 0.87 | 15.28 | 0.05 | 48 |  | |  |
| Plasma | 7 | -6.39 (-9.68, -3.11) | **<0.001** | 9.44 | 0.15 | 36 |  | |  |
| Basal condition |  |  |  |  |  |  | 0.44 | |  |
| Healthy | 3 | -5.67 (-14.07, 2.73) | 0.19 | 9.59 | 0.01 | 79 |  | |  |
| CVD risk | 13 | -2.2 (-5.05, 0.65) | 0.13 | 28.87 | 0 | 58 |  | |  |
| Control intervention |  |  |  |  |  |  | 0.28 | |  |
| Placebo | 11 | -4 (-6.3, -1.71) | **<0.001** | 9.52 | 0.48 | 0 |  | |  |
| Others | 5 | -0.81 (-6.18, 4.57) | 0.77 | 31.96 | 0 | 87 |  | |  |
| Baseline level |  |  |  |  |  |  | 0.27 | |  |
| <130 mg/dL | 9 | -1.54 (-4.89, 1.81) | 0.37 | 18.02 | 0.02 | 56 |  | |  |
| >130 mg/dL | 7 | -4.86 (-9.76, 0.04) | 0.05 | 23.49 | 0 | 74 |  | |  |
| **High-density lipoproteins** | | | | | | | |  |  |
| Overall | 17 | -1.08 (-1.99, -0.18) | **0.02** | 44.18 | 0 | 64 |  | |  |
| Treatment intervention |  |  |  |  |  |  | 0.95 | |  |
| Whole apple | 6 | -1.01 (-2.99, 0.97) | 0.32 | 30.7 | 0 | 84 |  | |  |
| Polyphenol | 9 | -0.72 (-1.55, 0.11) | 0.09 | 9.21 | 0.32 | 13 |  | |  |
| Apple pectin | 2 | -0.23 (-6.84, 6.38) | 0.95 | 2.67 | 0.1 | 63 |  | |  |
| Design |  |  |  |  |  |  | 0.2 | |  |
| Crossover RCT | 7 | -1.83 (-3.54, -0.11) | **0.04** | 31.25 | 0 | 81 |  | |  |
| Parallel RCT | 10 | -0.68 (-1.09, -0.28) | **<0.001** | 7.14 | 0.62 | 0 |  | |  |
| Sample type |  |  |  |  |  |  | 0.86 | |  |
| Serum | 10 | -0.88 (-1.5, -0.26) | **0.01** | 9.68 | 0.38 | 7 |  | |  |
| Plasma | 7 | -1.07 (-3.15, 1.01) | 0.31 | 33.21 | 0 | 82 |  | |  |
| Basal condition |  |  |  |  |  |  | 0.89 | |  |
| Healthy | 4 | -0.91 (-4.39, 2.57) | 0.61 | 11.75 | 0.01 | 74 |  | |  |
| CVD risk | 13 | -0.65 (-1.21, -0.09) | **0.02** | 13.92 | 0.31 | 14 |  | |  |
| Control intervention |  |  |  |  |  |  | 0.2 | |  |
| Placebo | 11 | -0.47 (-1.41, 0.47) | 0.32 | 6.95 | 0.73 | 0 |  | |  |
| Others | 6 | -1.64 (-3.17, -0.11) | **0.04** | 36.1 | 0 | 86 |  | |  |
| Baseline level |  |  |  |  |  |  | 0.33 | |  |
| <50 mg/dL | 6 | -0.7 (-1.11, -0.28) | **<0.001** | 3.91 | 0.56 | 0 |  | |  |
| >50 mg/dL | 11 | -1.42 (-2.82, -0.02) | **0.05** | 35.89 | 0 | 72 |  | |  |
| **Triglycerides** | | | | | | | |  |  |
| Overall | 17 | -1.62 (-6.82, 3.57) | 0.54 | 35.72 | 0 | 55 |  | |  |
| Treatment intervention |  |  |  |  |  |  | 0.8 | |  |
| Whole apple | 6 | -3.03 (-10.52, 4.45) | 0.43 | 11.24 | 0.05 | 56 |  | |  |
| Polyphenol | 9 | -0.67 (-9.35, 8.02) | 0.88 | 23.56 | 0 | 66 |  | |  |
| Apple pectin | 2 | 2.37 (-12.68, 17.42) | 0.76 | 0.01 | 0.94 | 0 |  | |  |
| Design |  |  |  |  |  |  | 0.27 | |  |
| Crossover RCT | 7 | -4.2 (-11.65, 3.25) | 0.27 | 22.64 | 0 | 74 |  | |  |
| Parallel RCT | 10 | 1.26 (-4.94, 7.45) | 0.69 | 9.72 | 0.37 | 7 |  | |  |
| Sample type |  |  |  |  |  |  | 0.95 | |  |
| Serum | 8 | -1.78 (-8.87, 5.32) | 0.62 | 12.28 | 0.09 | 43 |  | |  |
| Plasma | 9 | -1.47 (-9.43, 6.49) | 0.72 | 23.41 | 0 | 66 |  | |  |
| Basal condition |  |  |  |  |  |  | 0.85 | |  |
| Healthy | 3 | -0.74 (-7.22, 5.75) | 0.82 | 1.07 | 0.58 | 0 |  | |  |
| CVD risk | 14 | -1.64 (-8.01, 4.72) | 0.61 | 33.63 | 0 | 61 |  | |  |
| Control intervention |  |  |  |  |  |  | 0.61 | |  |
| Placebo | 11 | -2.66 (-10.78, 5.45) | 0.52 | 25.37 | 0 | 61 |  | |  |
| Others | 6 | -0.12 (-5.68, 5.43) | 0.97 | 7.04 | 0.22 | 29 |  | |  |
| Baseline level |  |  |  |  |  |  | 0.04 | |  |
| <150 mg/dL | 15 | -2.87 (-8.02, 2.28) | 0.28 | 29.61 | 0.01 | 53 |  | |  |
| >150 mg/dL | 2 | 12.58 (-1, 26.17) | 0.07 | 0.32 | 0.57 | 0 |  | |  |
| **Glucose** | | | | | | | |  |  |
| Overall | 12 | 0.43 (-0.99, 1.85) | 0.56 | 47.65 | 0 | 77 |  | |  |
| Treatment intervention |  |  |  |  |  |  | 0 | |  |
| Whole apple | 3 | 1.48 (-2.15, 5.11) | 0.42 | 7.88 | 0.02 | 75 |  | |  |
| Polyphenol | 6 | 0.49 (-1.18, 2.16) | 0.57 | 24.42 | 0 | 80 |  | |  |
| Apple pectin | 2 | 0.51 (-1.95, 2.98) | 0.68 | 0.01 | 0.91 | 0 |  | |  |
| Apple vinegar | 1 | -26.23 (-39.46, -13) | **<0.001** | NA | NA | NA |  | |  |
| Design |  |  |  |  |  |  | 0.82 | |  |
| Crossover RCT | 5 | -0.33 (-0.93, 0.26) | 0.27 | 2.88 | 0.58 | 0 |  | |  |
| Parallel RCT | 7 | 0.14 (-3.95, 4.22) | 0.95 | 33.85 | 0 | 82 |  | |  |
| Sample type |  |  |  |  |  |  | 0.8 | |  |
| Serum | 6 | 0.44 (-2.59, 3.48) | 0.77 | 45.04 | 0 | 89 |  | |  |
| Plasma | 6 | 0.04 (-0.68, 0.76) | 0.91 | 2.6 | 0.76 | 0 |  | |  |
| Basal condition |  |  |  |  |  |  | 0.89 | |  |
| Healthy | 1 | 0.7 (-3.31, 4.71) | 0.73 | NA | NA | NA |  | |  |
| CVD risk | 11 | 0.41 (-1.09, 1.91) | 0.6 | 47.53 | 0 | 79 |  | |  |
| Control intervention |  |  |  |  |  |  | 0.18 | |  |
| Placebo | 9 | 0.97 (-0.79, 2.74) | 0.28 | 32.45 | 0 | 75 |  | |  |
| Others | 3 | -1.99 (-5.95, 1.97) | 0.32 | 15.04 | 0 | 87 |  | |  |
| Baseline level |  |  |  |  |  |  | 0 | |  |
| <130 mg/dL | 11 | 0.63 (-0.58, 1.83) | 0.31 | 32.5 | 0 | 69 |  | |  |
| >130 mg/dL | 1 | -26.23 (-39.46, -13) | **<0.001** | NA | NA | NA |  | |  |
| **Insulin** | | | | | | | |  |  |
| Overall | 9 | -0.31 (-0.62, 0.01) | 0.06 | 7.19 | 0.52 | 0 |  | |  |
| Treatment intervention |  |  |  |  |  |  | 0.88 | |  |
| Whole apple | 4 | -0.46 (-0.87, -0.04) | **0.03** | 2.5 | 0.48 | 0 |  | |  |
| Polyphenol | 2 | -0.06 (-1.03, 0.9) | 0.9 | 2.76 | 0.1 | 64 |  | |  |
| Apple pectin | 2 | -0.51 (-1.62, 0.59) | 0.36 | 0 | 0.95 | 0 |  | |  |
| Apple vinegar | 1 | -0.05 (-2.36, 2.26) | 0.97 | NA | NA | NA |  | |  |
| Design |  |  |  |  |  |  | 0.77 | |  |
| Crossover RCT | 3 | -0.23 (-0.83, 0.37) | 0.46 | 5.04 | 0.08 | 60 |  | |  |
| Parallel RCT | 6 | -0.36 (-0.99, 0.28) | 0.27 | 2.12 | 0.83 | 0 |  | |  |
| Sample type |  |  |  |  |  |  | 0.02 | |  |
| Serum | 6 | -0.52 (-0.88, -0.15) | **0.01** | 1.04 | 0.96 | 0 |  | |  |
| Plasma | 3 | 0.39 (-0.27, 1.05) | 0.25 | 0.65 | 0.72 | 0 |  | |  |
| Basal condition |  |  |  |  |  |  | 0.81 | |  |
| Healthy | 2 | -0.36 (-1.06, 0.35) | 0.32 | 0.03 | 0.86 | 0 |  | |  |
| CVD risk | 7 | -0.26 (-0.68, 0.17) | 0.24 | 7.14 | 0.31 | 16 |  | |  |
| Control intervention |  |  |  |  |  |  | 0.91 | |  |
| Placebo | 7 | -0.27 (-0.7, 0.16) | 0.22 | 7.14 | 0.31 | 16 |  | |  |
| Others | 2 | -0.31 (-1.01, 0.38) | 0.37 | 0.05 | 0.81 | 0 |  | |  |
| **CRP** | | | | | | | |  |  |
| Overall | 6 | -0.02 (-0.38, 0.34) | 0.91 | 42.12 | 0 | 88 |  | |  |
| Treatment intervention |  |  |  |  |  |  | 0.6 | |  |
| Whole apple | 2 | 0.09 (-0.24, 0.43) | 0.58 | 0.11 | 0.73 | 0 |  | |  |
| Polyphenol | 4 | -0.06 (-0.53, 0.41) | 0.8 | 40.64 | 0 | 93 |  | |  |
| Design |  |  |  |  |  |  | 0.92 | |  |
| Crossover RCT | 3 | 0 (-0.56, 0.57) | 0.99 | 40.13 | 0 | 95 |  | |  |
| Parallel RCT | 3 | -0.03 (-0.31, 0.25) | 0.83 | 1.74 | 0.42 | 0 |  | |  |
| Sample type |  |  |  |  |  |  | 0 | |  |
| Serum | 4 | 0.23 (0.06, 0.39) | **0.01** | 3.36 | 0.34 | 11 |  | |  |
| Plasma | 2 | -0.47 (-0.64, -0.31) | **<0.001** | 0.62 | 0.43 | 0 |  | |  |
| Basal condition |  |  |  |  |  |  | 0.5 | |  |
| Healthy | 1 | 0.12 (-0.13, 0.37) | 0.33 | NA | NA | NA |  | |  |
| CVD risk | 5 | -0.05 (-0.5, 0.39) | 0.81 | 38.42 | 0 | 90 |  | |  |
| Control intervention |  |  |  |  |  |  | 0 | |  |
| Placebo | 3 | 0.24 (0.05, 0.43) | **0.01** | 2.82 | 0.24 | 29 |  | |  |
| Others | 3 | -0.4 (-0.63, -0.16) | **<0.001** | 2.59 | 0.27 | 23 |  | |  |
| Baseline level |  |  |  |  |  |  | 0.54 | |  |
| <10 mg/dL | 5 | -0.05 (-0.47, 0.36) | 0.81 | 40.76 | 0 | 90 |  | |  |
| >10 mg/dL | 1 | 0.13 (-0.26, 0.52) | 0.52 | NA | NA | NA |  | |  |
| **Diastolic blood pressure** | | | | | | | |  |  |
| Overall | 10 | -0.06 (-1.21, 1.1) | 0.92 | 16.81 | 0.05 | 46 |  | |  |
| Treatment intervention |  |  |  |  |  |  | 0.24 | |  |
| Whole apple | 3 | -0.08 (-3.72, 3.56) | 0.97 | 9.27 | 0.01 | 78 |  | |  |
| Polyphenol | 6 | -0.36 (-1.27, 0.55) | 0.44 | 4.62 | 0.46 | 0 |  | |  |
| Apple vinegar | 1 | 3.67 (-0.95, 8.29) | 0.12 | NA | NA | NA |  | |  |
| Design |  |  |  |  |  |  | 0.01 | |  |
| Crossover RCT | 6 | -0.79 (-1.64, 0.06) | 0.07 | 4.29 | 0.51 | 0 |  | |  |
| Parallel RCT | 4 | 2.51 (0.26, 4.75) | **0.03** | 3.55 | 0.31 | 15 |  | |  |
| Basal condition |  |  |  |  |  |  | 0.05 | |  |
| Healthy | 1 | -2.96 (-5.97, 0.05) | 0.05 | NA | NA | NA |  | |  |
| CVD risk | 9 | 0.19 (-0.95, 1.33) | 0.74 | 13.54 | 0.09 | 41 |  | |  |
| Control intervention |  |  |  |  |  |  | 1 | |  |
| Placebo | 6 | -0.04 (-1.51, 1.44) | 0.96 | 9.89 | 0.08 | 49 |  | |  |
| Others | 4 | -0.04 (-2.27, 2.2) | 0.97 | 6.85 | 0.08 | 56 |  | |  |
| Baseline level |  |  |  |  |  |  | 0.72 | |  |
| <80 mg/dL | 8 | -0.08 (-1.4, 1.24) | 0.9 | 13.09 | 0.07 | 47 |  | |  |
| >80 mg/dL | 2 | 0.78 (-3.79, 5.35) | 0.74 | 3.6 | 0.06 | 72 |  | |  |
| **Systolic blood pressure** | | | | | | | |  |  |
| Overall | 10 | 0.31 (-1.47, 2.09) | 0.73 | 16.12 | 0.06 | 44 |  | |  |
| Treatment intervention |  |  |  |  |  |  | 0.62 | |  |
| Whole apple | 3 | -0.92 (-5.24, 3.4) | 0.68 | 6.27 | 0.04 | 68 |  | |  |
| Polyphenol | 6 | 0.73 (-1.08, 2.54) | 0.43 | 6.82 | 0.23 | 27 |  | |  |
| Apple vinegar | 1 | 4.02 (-5.92, 13.96) | 0.43 | NA | NA | NA |  | |  |
| Design |  |  |  |  |  |  | 0.03 | |  |
| Crossover RCT | 6 | -0.65 (-2.57, 1.28) | 0.51 | 9.41 | 0.09 | 47 |  | |  |
| Parallel RCT | 4 | 3.11 (0.27, 5.96) | **0.03** | 0.63 | 0.89 | 0 |  | |  |
| Basal condition |  |  |  |  |  |  | 0.02 | |  |
| Healthy | 1 | -3.93 (-7.6, -0.26) | **0.04** | NA | NA | NA |  | |  |
| CVD risk | 9 | 0.75 (-0.93, 2.42) | 0.38 | 11.45 | 0.18 | 30 |  | |  |
| Control intervention |  |  |  |  |  |  | 0.98 | |  |
| Placebo | 6 | 0.39 (-1.82, 2.59) | 0.73 | 9.15 | 0.1 | 45 |  | |  |
| Others | 4 | 0.34 (-3.29, 3.97) | 0.86 | 6.97 | 0.07 | 57 |  | |  |
| Baseline level |  |  |  |  |  |  | 0.2 | |  |
| <120 mmHg | 2 | 2.31 (-1.08, 5.7) | 0.18 | 1.14 | 0.28 | 13 |  | |  |
| >120 mmHg | 8 | -0.22 (-2.16, 1.73) | 0.83 | 12.36 | 0.09 | 43 |  | |  |
| **^*^** Bold value indicates the statistical significance. | | | | | | | | |  |

**Supplementary Table 6**. Sensitivity analysis with correlation imputation of 0.2.

| **Study group** | **Trials** | **Estimate effect** | | **Heterogeneity** | | | |  |
| --- | --- | --- | --- | --- | --- | --- | --- | --- |
|  |  | **Weighted mean difference (95% CI)** | **p-effect^*^** | **Q statistic** | **p-within group** | **I^2^%** | **p-between group** | |
| **Total cholesterol** | | | | | | | | |
| Overall | 15 | -1.92 (-4.52, 0.67) | 0.15 | 10.04 | 0.76 | 0 |  | |
| Treatment intervention |  |  |  |  |  |  | 0.15 | |
| Whole apple | 4 | -0.43 (-3.79, 2.93) | 0.8 | 3.02 | 0.39 | 1 |  | |
| Polyphenol | 9 | -3.83 (-8.22, 0.57) | 0.09 | 3.1 | 0.93 | 0 |  | |
| Apple pectin | 2 | -15.22 (-32.42, 1.97) | 0.08 | 0.06 | 0.81 | 0 |  | |
| Design |  |  |  |  |  |  | 0.34 | |
| Crossover RCT | 6 | -3.91 (-8.73, 0.92) | 0.11 | 2.65 | 0.75 | 0 |  | |
| Parallel RCT | 9 | -1.12 (-4.19, 1.95) | 0.47 | 6.48 | 0.59 | 0 |  | |
| Sample type |  |  |  |  |  |  | 0.15 | |
| Serum | 9 | -1.09 (-3.93, 1.74) | 0.45 | 5.3 | 0.72 | 0 |  | |
| Plasma | 6 | -6.21 (-12.64, 0.22) | 0.06 | 2.7 | 0.75 | 0 |  | |
| Basal condition |  |  |  |  |  |  | 0.6 | |
| Healthy | 2 | -5.31 (-18.19, 7.57) | 0.42 | 0.87 | 0.35 | 0 |  | |
| CVD risk | 13 | -1.78 (-4.43, 0.87) | 0.19 | 8.89 | 0.71 | 0 |  | |
| Control intervention |  |  |  |  |  |  | 0.1 | |
| Placebo | 11 | -5.14 (-9.74, -0.54) | **0.03** | 4.6 | 0.92 | 0 |  | |
| Others | 4 | -0.42 (-3.56, 2.72) | 0.79 | 2.69 | 0.44 | 0 |  | |
| Baseline level |  |  |  |  |  |  | 0.85 | |
| <200 mg/dL | 4 | -0.73 (-13.69, 12.24) | 0.91 | 0.97 | 0.81 | 0 |  | |
| >200 mg/dL | 11 | -1.97 (-4.62, 0.67) | 0.14 | 9.03 | 0.53 | 0 |  | |
| **Low-density lipoproteins** | | | | | | | | |
| Overall | 16 | -1.15 (-3.38, 1.08) | 0.31 | 15.9 | 0.39 | 6 |  | |
| Treatment intervention |  |  |  |  |  |  | 0.33 | |
| Whole apple | 5 | -1.69 (-6.22, 2.84) | 0.47 | 6.02 | 0.2 | 34 |  | |
| Polyphenol | 9 | -2.45 (-6.34, 1.44) | 0.22 | 5.09 | 0.75 | 0 |  | |
| Apple pectin | 2 | -13.92 (-29.45, 1.61) | 0.08 | 0 | 0.99 | 0 |  | |
| Design |  |  |  |  |  |  | 0.09 | |
| Crossover RCT | 7 | -3.22 (-7.05, 0.61) | 0.1 | 6.21 | 0.4 | 3 |  | |
| Parallel RCT | 9 | 0.55 (-1.39, 2.5) | 0.58 | 6.56 | 0.59 | 0 |  | |
| Sample type |  |  |  |  |  |  | 0.01 | |
| Serum | 9 | 0.73 (-1.13, 2.59) | 0.44 | 5.61 | 0.69 | 0 |  | |
| Plasma | 7 | -6.33 (-10.96, -1.7) | **0.01** | 2.59 | 0.86 | 0 |  | |
| Basal condition |  |  |  |  |  |  | 0.15 | |
| Healthy | 3 | -5.69 (-13.5, 2.11) | 0.15 | 2.56 | 0.28 | 22 |  | |
| CVD risk | 13 | 0.22 (-1.58, 2.02) | 0.81 | 9.88 | 0.63 | 0 |  | |
| Control intervention |  |  |  |  |  |  | 0.37 | |
| Placebo | 11 | -4.08 (-8.09, -0.08) | **0.05** | 2.61 | 0.99 | 0 |  | |
| Others | 5 | -1.08 (-6.34, 4.18) | 0.69 | 8.98 | 0.06 | 55 |  | |
| Baseline level |  |  |  |  |  |  | 0.08 | |
| <130 mg/dL | 9 | 0.38 (-1.49, 2.24) | 0.69 | 6.87 | 0.55 | 0 |  | |
| >130 mg/dL | 7 | -4.05 (-8.62, 0.53) | 0.08 | 5.96 | 0.43 | 0 |  | |
| **High-density lipoproteins** | | | | | | | | |
| Overall | 17 | -0.84 (-1.24, -0.44) | **<0.001** | 11.84 | 0.76 | 0 |  | |
| Treatment intervention |  |  |  |  |  |  | 0.83 | |
| Whole apple | 6 | -1.31 (-3.13, 0.51) | 0.16 | 8.43 | 0.13 | 41 |  | |
| Polyphenol | 9 | -0.62 (-1.83, 0.59) | 0.32 | 2.55 | 0.96 | 0 |  | |
| Apple pectin | 2 | -0.8 (-8.33, 6.74) | 0.84 | 0.71 | 0.4 | 0 |  | |
| Design |  |  |  |  |  |  | 0.22 | |
| Crossover RCT | 7 | -1.8 (-3.42, -0.18) | 0.03 | 7.84 | 0.25 | 24 |  | |
| Parallel RCT | 10 | -0.75 (-1.17, -0.33) | **<0.001** | 2.31 | 0.99 | 0 |  | |
| Sample type |  |  |  |  |  |  | 0.59 | |
| Serum | 10 | -0.79 (-1.21, -0.38) | **<0.001** | 2.94 | 0.97 | 0 |  | |
| Plasma | 7 | -1.35 (-3.35, 0.64) | 0.18 | 8.42 | 0.21 | 29 |  | |
| Basal condition |  |  |  |  |  |  | 0.41 | |
| Healthy | 4 | -2.11 (-5.28, 1.06) | 0.19 | 4.06 | 0.26 | 26 |  | |
| CVD risk | 13 | -0.76 (-1.17, -0.35) | **<0.001** | 3.72 | 0.99 | 0 |  | |
| Control intervention |  |  |  |  |  |  | 0.46 | |
| Placebo | 11 | -0.56 (-2.07, 0.95) | 0.47 | 2.13 | 1 | 0 |  | |
| Others | 6 | -1.35 (-2.78, 0.08) | 0.06 | 9.56 | 0.09 | 48 |  | |
| Baseline level |  |  |  |  |  |  | 0.37 | |
| <50 mg/dL | 6 | -0.77 (-1.19, -0.34) | **<0.001** | 1.24 | 0.94 | 0 |  | |
| >50 mg/dL | 11 | -1.31 (-2.41, -0.21) | **0.02** | 9.78 | 0.46 | 0 |  | |
| **Triglycerides** | | | | | | | | |
| Overall | 17 | -1.75 (-7.01, 3.51) | 0.51 | 14.22 | 0.58 | 0 |  | |
| Treatment intervention |  |  |  |  |  |  | 0.93 | |
| Whole apple | 6 | -1.41 (-10.66, 7.84) | 0.76 | 6.85 | 0.23 | 27 |  | |
| Polyphenol | 9 | -2.72 (-10.37, 4.93) | 0.49 | 7.19 | 0.52 | 0 |  | |
| Apple pectin | 2 | 2.33 (-26.1, 30.76) | 0.87 | 0 | 0.96 | 0 |  | |
| Design |  |  |  |  |  |  | 0.15 | |
| Crossover RCT | 7 | -4.84 (-11.9, 2.21) | 0.18 | 6.58 | 0.36 | 9 |  | |
| Parallel RCT | 10 | 3.32 (-5.15, 11.79) | 0.44 | 5.4 | 0.8 | 0 |  | |
| Sample type |  |  |  |  |  |  | 0.53 | |
| Serum | 10 | -3.1 (-11.16, 4.96) | 0.45 | 12.34 | 0.19 | 27 |  | |
| Plasma | 7 | 0.76 (-8.05, 9.57) | 0.87 | 1.39 | 0.97 | 0 |  | |
| Basal condition |  |  |  |  |  |  | 0.9 | |
| Healthy | 3 | -1.08 (-13.12, 10.97) | 0.86 | 0.29 | 0.86 | 0 |  | |
| CVD risk | 14 | -1.9 (-8.06, 4.25) | 0.54 | 13.91 | 0.38 | 7 |  | |
| Control intervention |  |  |  |  |  |  | 0.16 | |
| Placebo | 11 | -5.93 (-13.81, 1.95) | 0.14 | 7.67 | 0.66 | 0 |  | |
| Others | 6 | 1.6 (-5.46, 8.67) | 0.66 | 4.6 | 0.47 | 0 |  | |
| Baseline level |  |  |  |  |  |  | 0.02 | |
| <150 mg/dL | 15 | -4.22 (-9.89, 1.45) | 0.14 | 8.9 | 0.84 | 0 |  | |
| >150 mg/dL | 2 | 13.42 (-0.64, 27.47) | 0.06 | 0.11 | 0.74 | 0 |  | |
| **Glucose** | | | | | | | | |
| Overall | 12 | 0.21 (-1.28, 1.7) | 0.78 | 16.17 | 0.13 | 32 |  | |
| Treatment intervention |  |  |  |  |  |  | 0.22 | |
| Whole apple | 3 | 1.05 (-2.49, 4.59) | 0.56 | 2.8 | 0.25 | 28 |  | |
| Polyphenol | 6 | 0.1 (-1.73, 1.92) | 0.92 | 8.99 | 0.11 | 44 |  | |
| Apple pectin | 2 | 0.51 (-4.24, 5.26) | 0.83 | 0 | 0.95 | 0 |  | |
| Apple vinegar | 1 | -26.23 (-51.45, -1.01) | **0.04** | 0 | 1 | 0 |  | |
| Design |  |  |  |  |  |  | 0.49 | |
| Crossover RCT | 5 | -0.34 (-1.49, 0.81) | 0.56 | 0.78 | 0.94 | 0 |  | |
| Parallel RCT | 7 | 1.19 (-3.04, 5.42) | 0.58 | 12.46 | 0.05 | 52 |  | |
| Sample type |  |  |  |  |  |  | 0.58 | |
| Serum | 6 | 0.87 (-2.18, 3.91) | 0.58 | 14 | 0.02 | 64 |  | |
| Plasma | 6 | -0.07 (-1.49, 1.34) | 0.92 | 2.1 | 0.84 | 0 |  | |
| Basal condition |  |  |  |  |  |  | 0.9 | |
| Healthy | 1 | 0.7 (-7.01, 8.41) | 0.86 | NA | NA | NA |  | |
| CVD risk | 11 | 0.21 (-1.37, 1.8) | 0.79 | 16.15 | 0.1 | 38 |  | |
| Control intervention |  |  |  |  |  |  | 0.63 | |
| Placebo | 9 | 0.51 (-1.43, 2.45) | 0.61 | 11.96 | 0.15 | 33 |  | |
| Others | 3 | -0.44 (-3.74, 2.86) | 0.79 | 4.14 | 0.13 | 52 |  | |
| Baseline level |  |  |  |  |  |  | 0.04 | |
| <130 mg/dL | 11 | 0.2 (-1.07, 1.46) | 0.76 | 11.99 | 0.29 | 17 |  | |
| >130 mg/dL | 1 | -26.23 (-51.45, -1.01) | **0.04** | 0 | 1 | 0 |  | |
| **Insulin** | | | | | | | | |
| Overall | 9 | -0.27 (-0.84, 0.31) | 0.36 | 2.37 | 0.97 | 0 |  | |
| Treatment intervention |  |  |  |  |  |  | 0.85 | |
| Whole apple | 4 | -0.45 (-1.27, 0.36) | 0.28 | 0.68 | 0.88 | 0 |  | |
| Polyphenol | 2 | 0.08 (-0.89, 1.04) | 0.87 | 0.87 | 0.35 | 0 |  | |
| Apple pectin | 2 | -0.55 (-2.18, 1.08) | 0.51 | 0 | 0.95 | 0 |  | |
| Apple vinegar | 1 | -0.05 (-4.21, 4.11) | 0.98 | NA | NA | NA |  | |
| Design |  |  |  |  |  |  | 0.77 | |
| Crossover RCT | 3 | -0.21 (-0.9, 0.47) | 0.54 | 1.67 | 0.43 | 0 |  | |
| Parallel RCT | 6 | -0.4 (-1.49, 0.68) | 0.47 | 0.61 | 0.99 | 0 |  | |
| Sample type |  |  |  |  |  |  | 0.24 | |
| Serum | 6 | -0.51 (-1.21, 0.19) | 0.16 | 0.32 | 1 | 0 |  | |
| Plasma | 3 | 0.23 (-0.78, 1.24) | 0.66 | 0.66 | 0.72 | 0 |  | |
| Basal condition |  |  |  |  |  |  | 0.79 | |
| Healthy | 2 | -0.41 (-1.62, 0.79) | 0.5 | 0.04 | 0.85 | 0 |  | |
| CVD risk | 7 | -0.22 (-0.88, 0.43) | 0.5 | 2.26 | 0.89 | 0 |  | |
| Control intervention |  |  |  |  |  |  | 0.95 | |
| Placebo | 7 | -0.26 (-0.9, 0.38) | 0.43 | 2.35 | 0.89 | 0 |  | |
| Others | 2 | -0.31 (-1.65, 1.03) | 0.65 | 0.02 | 0.9 | 0 |  | |
| **CRP** | | | | | | | | |
| Overall | 6 | -0.04 (-0.32, 0.24) | 0.76 | 11.07 | 0.05 | 55 |  | |
| Treatment intervention |  |  |  |  |  |  | 0.65 | |
| Whole apple | 2 | 0.05 (-0.29, 0.39) | 0.77 | 0.03 | 0.86 | 0 |  | |
| Polyphenol | 4 | -0.07 (-0.48, 0.34) | 0.73 | 10.23 | 0.02 | 71 |  | |
| Design |  |  |  |  |  |  | 0.93 | |
| Crossover RCT | 3 | -0.01 (-0.58, 0.55) | 0.97 | 10.03 | 0.01 | 80 |  | |
| Parallel RCT | 3 | -0.04 (-0.32, 0.23) | 0.77 | 0.91 | 0.63 | 0 |  | |
| Sample type |  |  |  |  |  |  | 0 | |
| Serum | 4 | 0.16 (-0.08, 0.4) | 0.2 | 1.43 | 0.7 | 0 |  | |
| Plasma | 2 | -0.4 (-0.69, -0.12) | **<0.001** | 0.78 | 0.38 | 0 |  | |
| Basal condition |  |  |  |  |  |  | 0.52 | |
| Healthy | 1 | 0.12 (-0.37, 0.62) | 0.63 | NA | NA | NA |  | |
| CVD risk | 5 | -0.07 (-0.4, 0.25) | 0.67 | 10.34 | 0.04 | 61 |  | |
| Control intervention |  |  |  |  |  |  | 0.01 | |
| Placebo | 3 | 0.18 (-0.08, 0.44) | 0.17 | 1.16 | 0.56 | 0 |  | |
| Others | 3 | -0.34 (-0.6, -0.07) | **0.01** | 2.06 | 0.36 | 3 |  | |
| Baseline level |  |  |  |  |  |  | 0.62 | |
| <10 mg/dL | 5 | -0.06 (-0.41, 0.28) | 0.71 | 10.38 | 0.03 | 61 |  | |
| >10 mg/dL | 1 | 0.07 (-0.32, 0.46) | 0.73 | NA | NA | NA |  | |
| **Diastolic blood pressure** | | | | | | | | |
| Overall | 10 | -0.52 (-1.66, 0.61) | 0.37 | 9.31 | 0.41 | 3 |  | |
| Treatment intervention |  |  |  |  |  |  | 0.2 | |
| Whole apple | 3 | -1.06 (-4.46, 2.34) | 0.54 | 3.35 | 0.19 | 40 |  | |
| Polyphenol | 6 | -0.55 (-1.81, 0.71) | 0.39 | 1.93 | 0.86 | 0 |  | |
| Apple vinegar | 1 | 3.67 (-0.95, 8.29) | 0.12 | NA | NA | NA |  | |
| Design |  |  |  |  |  |  | 0.05 | |
| Crossover RCT | 6 | -0.97 (-2.12, 0.19) | 0.1 | 2.7 | 0.75 | 0 |  | |
| Parallel RCT | 4 | 2.35 (-0.69, 5.39) | 0.13 | 2.62 | 0.45 | 0 |  | |
| Basal condition |  |  |  |  |  |  | 0.09 | |
| Healthy | 1 | -2.96 (-5.97, 0.05) | 0.05 | NA | NA | NA |  | |
| CVD risk | 9 | -0.19 (-1.35, 0.97) | 0.75 | 6.48 | 0.59 | 0 |  | |
| Control intervention |  |  |  |  |  |  | 0.72 | |
| Placebo | 6 | -0.6 (-1.9, 0.7) | 0.37 | 3.1 | 0.69 | 0 |  | |
| Others | 4 | -0.01 (-2.91, 2.9) | 0.99 | 6.2 | 0.1 | 52 |  | |
| Baseline level |  |  |  |  |  |  | 0.47 | |
| <80 mg/dL | 8 | -0.75 (-1.93, 0.42) | 0.21 | 5.89 | 0.55 | 0 |  | |
| >80 mg/dL | 2 | 1.02 (-3.63, 5.66) | 0.67 | 2.66 | 0.1 | 62 |  | |
| **Systolic blood pressure** | | | | | | | | |
| Overall | 10 | -1.13 (-2.76, 0.5) | 0.18 | 8.5 | 0.48 | 0 |  | |
| Treatment intervention |  |  |  |  |  |  | 0.36 | |
| Whole apple | 3 | -2.71 (-6.16, 0.74) | 0.12 | 2.17 | 0.34 | 8 |  | |
| Polyphenol | 6 | -0.64 (-2.6, 1.31) | 0.52 | 3.85 | 0.57 | 0 |  | |
| Apple vinegar | 1 | 4.02 (-5.92, 13.96) | 0.43 | NA | NA | NA |  | |
| Design |  |  |  |  |  |  | 0.05 | |
| Crossover RCT | 6 | -1.71 (-3.45, 0.03) | 0.05 | 4.57 | 0.47 | 0 |  | |
| Parallel RCT | 4 | 3.35 (-1.47, 8.17) | 0.17 | 0.19 | 0.98 | 0 |  | |
| Basal condition |  |  |  |  |  |  | 0.1 | |
| Healthy | 1 | -3.93 (-7.6, -0.26) | **0.04** | NA | NA | NA |  | |
| CVD risk | 9 | -0.44 (-2.26, 1.38) | 0.64 | 5.72 | 0.68 | 0 |  | |
| Control intervention |  |  |  |  |  |  | 0.73 | |
| Placebo | 6 | -1.02 (-3.02, 0.98) | 0.32 | 3.55 | 0.62 | 0 |  | |
| Others | 4 | -0.21 (-4.3, 3.88) | 0.92 | 4.92 | 0.18 | 39 |  | |
| Baseline level |  |  |  |  |  |  | 0.25 | |
| <120 mmHg | 2 | 2.17 (-3.65, 7.99) | 0.47 | 0.31 | 0.58 | 0 |  | |
| >120 mmHg | 8 | -1.41 (-3.11, 0.29) | 0.1 | 6.85 | 0.44 | 0 |  | |
| **^*^** Bold value indicates the statistical significance. | | | | | | | | |

**Supplementary Table 7.** Publication bias: Egger's regression test and Begg’s rank test (not-continuity corrected).

| P-value (2-tailed) | TC | HDL | LDL | TG | Glucose | DBP | SBP |
| --- | --- | --- | --- | --- | --- | --- | --- |
| Egger’s test | 0.18 | 0.71 | 0.06 | 0.24 | 0.90 | 0.36 | 0.01 |
| Begg’s test | 0.88 | 0.62 | 0.72 | 0.41 | 0.68 | 0.13 | 0.09 |

**Supplementary** **Table 8.** Whole evaluation outcomes of quality assessment.

| **Author** | **R** | **D** | **Mi** | **Me** | **S** | **O** | |
| --- | --- | --- | --- | --- | --- | --- | --- |
| Shoji et al., Japan, 2017 (1) | **+** | **+** | **+** | **+** | **+** | **+** | |
| Gasper et al., United Kingdom, 2014 (2) | **?** | **?** | **+** | **+** | **+** | **?** | |
| Chai et al., United States, 2012 (3) | **?** | **-** | **+** | **+** | **+** | **-** | |
| Cicero et al., Italy, 2017 (4) | **+** | **-** | **+** | **+** | **+** | **-** | |
| Vafa et al., Iran, 2011 (5) | **?** | **-** | **+** | **+** | **+** | **-** | |
| Eisner et al., USA, 2020 (6) | **-** | **-** | **+** | **+** | **?** | **-** | |
| Akazome et al., Japan, 2010 (7) | **?** | **+** | **+** | **+** | **+** | **?** | |
| Bondonno et al., Australia, 2018 (8) | **+** | **+** | **+** | **+** | **+** | **+** | |
| Sirtori et al., Italy, 2012 (9) | **?** | **+** | **+** | **+** | **+** | **?** | |
| Ravn-Haren et al., Denmark, 2013 (10) | **+** | **-** | **+** | **+** | **+** | **-** | |
| Hollands et al., United Kingdom, 2018 (11) | **+** | **+** | **+** | **+** | **+** | **+** | |
| Barth et al., Germany, 2012 (12) | **?** | **+** | **+** | **+** | **+** | **?** | |
| Saarenhovi et al., Finland, 2017 (13) | **?** | **+** | **+** | **+** | **+** | **?** | |
| Auclair et al., France, 2010 (14) | **?** | **-** | **+** | **+** | **+** | **-** | |
| Koutsos et al., Italy, 2019 (15) | **+** | **+** | **+** | **+** | **+** | **+** | |
| Velliquette et al., USA, 2015 (16) | **?** | **-** | **+** | **+** | **+** | **-** | |
| Liddle et al., Canada, 2021 (17) | **?** | **+** | **+** | **+** | **+** | **?** | |
| Gheflati et al., Iran, 2019 (18) | **?** | **-** | **+** | **+** | **+** | **-** | |
| R: Randomisation process, D: Deviations from intended intervention, Mi: Missing outcome data, Me: Measurement of the outcome, S: Selection of the reported results, O: Overall  +: Low risk, ?: Some concerns, -: High risk | | | | | | |  |

**Supplementary** **Table 9.** Evidence profile of Grading of Recommendations Assessment, Development, and Evaluation (GRADE) assessment.

| Quality assessment | | | | | | Number of participants | Effect size | Quality of Evidence |
| --- | --- | --- | --- | --- | --- | --- | --- | --- |
| *Number of studies (Design)* | *Limitations (risk of bias)* | *Inconsistency* | *Indirectness* | *Imprecision* | *Publication bias* |  | *Mean difference (95% CI)* |  |
| Total cholesterol (mg/dL) | | | | | | | | |
| 15 (RCT) | not serious | not serious | serious ^c^ | not serious | not serious | 670 | -2.33 (-4.69, 0.03) | Moderate |
| Low-density lipoproteins (mg/dL) | | | | | | | | |
| 16 (RCT) | not serious | not serious | serious ^c^ | not serious | not serious | 693 | -2.6 (-5.38, 0.19) | Moderate |
| High-density lipoproteins (mg/dL) | | | | | | | | |
| 17 (RCT) | serious ^a^ | not serious | serious ^c^ | not serious | not serious | 793 | -1.01 (-1.71, -0.31) | Low |
| Triglycerides (mg/dL) | | | | | | | | |
| 17 (RCT) | not serious | not serious | serious ^c^ | not serious | not serious | 793 | -2.2 (-7.24, 2.85) | Moderate |
| Glucose (mg/dL) | | | | | | | | |
| 12 (RCT) | not serious | serious ^b^ | serious ^c^ | not serious | not serious | 553 | 0.34 (-1.16, 1.84) | Low |
| Insulin (µU/mL) | | | | | | | | |
| 9 (RCT) | not serious | not serious | serious ^c^ | serious ^d^ | not serious | 397 | -0.27 (-0.74, 0.21) | Low |
| C-reactive protein | | | | | | | | |
| 6 (RCT) | serious ^a^ | serious ^b^ | serious ^c^ | serious ^d^ | not serious | 326 | -0.03 (-0.34, 0.28) | Very low |
| Diastolic blood pressure (mmHg) | | | | | | | | |
| 10 (RCT) | not serious | not serious | serious ^c^ | not serious | not serious | 423 | -0.34 (-1.52, 0.84) | Moderate |
| Systolic blood pressure (mmHg) | | | | | | | | |
| 10 (RCT) | not serious | not serious | serious ^c^ | not serious | not serious ^e^ | 423 | -0.44 (-2.22, 1.33) | Moderate |
| ^a^ Downgraded, because removing studies graded as high risk of bias reversed significance of the result for HDL, and significant proportion of included studies has 'high risk' aspect for CRP.  ^b^ Downgraded, because of high *I^2^* values for glucose (*I^2^* = 53) and CRP (*I^2^* = 66).  ^c^ Downgraded, because there existed different basal conditions among participants, different formula among the interventions, and different types of control intervention.  ^d^ Downgraded, because of the low number of populations for insulin and CRP (less than 400).  ^e^ Not downgraded, because small studies reported outcomes at unfavored intervention although the tests of asymmetry reported a potential risk of publication bias; this can be observed in the funnel plot. | | | | | | | | |

**Reference**

1. Shoji T, Yamada M, Miura T, Nagashima K, Ogura K, Inagaki N, et al. Chronic administration of apple polyphenols ameliorates hyperglycaemia in high-normal and borderline subjects: A randomised, placebo-controlled trial. Diabetes Res Clin Pract. 2017;129:43-51.

2. Gasper A, Hollands W, Casgrain A, Saha S, Teucher B, Dainty JR, et al. Consumption of both low and high (-)-epicatechin apple puree attenuates platelet reactivity and increases plasma concentrations of nitric oxide metabolites: a randomized controlled trial. Arch Biochem Biophys. 2014;559:29-37.

3. Chai SC, Hooshmand S, Saadat RL, Payton ME, Brummel-Smith K, Arjmandi BH. Daily apple versus dried plum: impact on cardiovascular disease risk factors in postmenopausal women. J Acad Nutr Diet. 2012;112(8):1158-68.

4. Cicero AFG, Caliceti C, Fogacci F, Giovannini M, Calabria D, Colletti A, et al. Effect of apple polyphenols on vascular oxidative stress and endothelium function: a translational study. Molecular nutrition & food research. 2017;61(11).

5. Vafa MR, Haghighatjoo E, Shidfar F, Afshari S, Gohari MR, Ziaee A. Effects of apple consumption on lipid profile of hyperlipidemic and overweight men. Int J Prev Med. 2011;2(2):94-100.

6. Eisner A, Ramachandran P, Cabalbag C, Metti D, Shamloufard P, Kern M, et al. Effects of Dried Apple Consumption on Body Composition, Serum Lipid Profile, Glucose Regulation, and Inflammatory Markers in Overweight and Obese Children. Journal of medicinal food. 2020;23(3):242-9.

7. Akazome Y, Kametani N, Kanda T, Shimasaki H, Kobayashi S. Evaluation of safety of excessive intake and efficacy of long-term intake of beverages containing apple polyphenols. Journal of oleo science. 2010;59(6):321-38.

8. Bondonno NP, Bondonno CP, Blekkenhorst LC, Considine MJ, Maghzal G, Stocker R, et al. Flavonoid-Rich Apple Improves Endothelial Function in Individuals at Risk for Cardiovascular Disease: A Randomized Controlled Clinical Trial. Molecular nutrition & food research. 2018;62(3).

9. Sirtori CR, Triolo M, Bosisio R, Bondioli A, Calabresi L, De Vergori V, et al. Hypocholesterolaemic effects of lupin protein and pea protein/fibre combinations in moderately hypercholesterolaemic individuals. Br J Nutr. 2012;107(8):1176-83.

10. Ravn-Haren G, Dragsted LO, Buch-Andersen T, Jensen EN, Jensen RI, Németh-Balogh M, et al. Intake of whole apples or clear apple juice has contrasting effects on plasma lipids in healthy volunteers. Eur J Nutr. 2013;52(8):1875-89.

11. Hollands WJ, Tapp H, Defernez M, Perez Moral N, Winterbone MS, Philo M, et al. Lack of acute or chronic effects of epicatechin-rich and procyanidin-rich apple extracts on blood pressure and cardiometabolic biomarkers in adults with moderately elevated blood pressure: a randomized, placebo-controlled crossover trial. Am J Clin Nutr. 2018;108(5):1006-14.

12. Barth SW, Koch TC, Watzl B, Dietrich H, Will F, Bub A. Moderate effects of apple juice consumption on obesity-related markers in obese men: impact of diet-gene interaction on body fat content. Eur J Nutr. 2012;51(7):841-50.

13. Saarenhovi M, Salo P, Scheinin M, Lehto J, Lovró Z, Tiihonen K, et al. The effect of an apple polyphenol extract rich in epicatechin and flavan-3-ol oligomers on brachial artery flow-mediated vasodilatory function in volunteers with elevated blood pressure. Nutr J. 2017;16(1):73.

14. Auclair S, Chironi G, Milenkovic D, Hollman PC, Renard CM, Mégnien JL, et al. The regular consumption of a polyphenol-rich apple does not influence endothelial function: a randomised double-blind trial in hypercholesterolemic adults. Eur J Clin Nutr. 2010;64(10):1158-65.

15. Koutsos A, Riccadonna S, Ulaszewska MM, Franceschi P, Trošt K, Galvin A, et al. Two apples a day lower serum cholesterol and improve cardiometabolic biomarkers in mildly hypercholesterolemic adults: a randomized, controlled, crossover trial. Am J Clin Nutr. 2020;111(2):307-18.

16. Velliquette RA, Grann K, Missler SR, Patterson J, Hu C, Gellenbeck KW, et al. Identification of a botanical inhibitor of intestinal diacylglyceride acyltransferase 1 activity via in vitro screening and a parallel, randomized, blinded, placebo-controlled clinical trial. Nutrition & metabolism. 2015;12:27.

17. Liddle DM, Lin X, Cox LC, Ward EM, Ansari R, Wright AJ, et al. Daily apple consumption reduces plasma and peripheral blood mononuclear cell–secreted inflammatory biomarkers in adults with overweight and obesity: a 6-week randomized, controlled, parallel-arm trial. The American Journal of Clinical Nutrition. 2021.

18. Gheflati A, Bashiri R, Ghadiri-Anari A, Reza JZ, Kord MT, Nadjarzadeh A. The effect of apple vinegar consumption on glycemic indices, blood pressure, oxidative stress, and homocysteine in patients with type 2 diabetes and dyslipidemia: A randomized controlled clinical trial. Clinical nutrition ESPEN. 2019;33:132-8.
